# The deubiquitinase OTUD3 plays a neuroprotective role by reducing ferroptosis induced by cerebral ischaemia reperfusion via stabilizing PLK1 via deubiquitination

**DOI:** 10.1002/ctm2.70347

**Published:** 2025-06-03

**Authors:** Jing Cheng, Qi Tian, Hao‐Ran Lu, Hong‐Xiang Jiang, Xiao‐Hong Qin, Yan‐Qin Fan, Zhi‐Biao Chen, Li‐Quan Wu

**Affiliations:** ^1^ Department of Neurosurgery Renmin Hospital of Wuhan University Wuhan China; ^2^ Division of Nephrology Renmin Hospital of Wuhan University Wuhan China

**Keywords:** deubiquitination, ischaemia‐reperfusion injury, neuroprotection, OTUD3, PLK1

## Abstract

Ischaemic stroke is one of the most common serious diseases observed in elderly people, which is caused by ischaemia‐reperfusion (I/R) injury. Ovarian tumour domain‐containing protein 3 (OTUD3) is a member of the ovarian tumour proteases (OTUs) family of deubiquitination enzymes located in the cytoplasm. We previously showed that the expression of OTUD3 in neurons was significantly reduced after cerebral I/R in mice. In addition, OTUD3 knockdown aggravated ferroptosis and brain damage following I/R in mice, and overexpression of OTUD3 reduced the mortality of cortical neurons in an oxygen glucose deprivation model (OGD/R). Co‐immunoprecipitation‐mass spectrometry analysis revealed that OTUD3 could bind to the amino acid sequence 35–305 of PLK1. Single‐cell sequencing results suggested that PLK1 expression was significantly reduced in mouse neurons after I/R injury. Similarly, reduced PLK1 expression was found in the cortical brain tissues of I/R mice and in the OGD/R‐stimulated primary cortical neurons of mice. In vitro experiments showed that OTUD3 overexpression led to the upregulation of PLK1 expression, and inhibition of PLK1 suppressed the inhibitory effect of OTUD3 overexpression on ferroptosis. Moreover, PLK1 positively regulated the PI3K/AKT signalling pathway in neurons after I/R injury, and inhibition of PI3K activity suppressed the inhibitory effect of PLK1 on ferroptosis. Ubiquitination experiments showed that OTUD3 modified PLK1 through deubiquitinating K48‐linked ubiquitination, thereby reducing its degradation by ubiquitination and stabilizing PLK1 expression. These results indicated that OTUD3 could upregulate PLK1 through deubiquitination modification, thereby activating the PI3K/AKT signalling pathway and reducing ferroptosis after cerebral I/R. Animal behavioural experiments and cellular methyl thiazolyl tetrazolium and lactate dehydrogenase experiments revealed that inhibition of PLK1 exacerbated brain damage after I/R in mice. Inhibition of OTUD3 deubiquitination enzyme activity attenuated the neuroprotective effect of OTUD3. In conclusion, our findings provide evidence that OTUD3 reduces ferroptosis by upregulating PLK1 expression through deubiquitination modification and exerts neuroprotective effects in cerebral I/R injury.

**Key points:**

- For the first time, it has been clarified that OTUD3 exerts neuroprotective effects in cerebral ischemia/reperfusion injury by deubiquitinating PLK1 to regulate the PI3K/AKT pathway and inhibit ferroptosis.
- The study first demonstrates that OTUD3 binds to the amino acid residues 35305 of PLK1 and deubiquitinates PLK1 (targeting K48‐linked ubiquitination), thereby reducing its degradation and stabilizing PLK1 protein expression.

## Graphical abstract

Ovarian tumour domain‐containing protein 3 (OTUD3) modified PLK1 through deubiquitinating K48‐linked ubiquitination in the amino acid sequence 35–305 of PLK1. Decreased expression of OTUD3 after cerebral ischaemia‐reperfusion resulted in increased PLK1 ubiquitination and decreased PLK1 protein expression. In turn, this inhibited the activation of the PI3K/AKT signalling pathway, reduced glutathione content, inhibited the activity of GPX4 and led to an increase in the generation of ROS, which ultimately contributed to the occurrence of ferroptosis in neuronal cells.

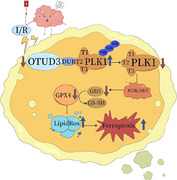

## 1 INTRODUCTION

Stroke is a cerebrovascular disease that is associated with high disability and mortality rates. It is a serious cerebral injury caused by bleeding due to rupture of cerebral blood vessels or insufficient cerebral blood supply due to occlusion of cerebral blood vessels. Among the different types of stroke, acute ischaemic stroke accounts for about 87% of cases.^1^, ^2^ Following a stroke, cerebral hypoxia and glucose deficiency are observed, which lead to neuronal death and neurological deficits, and prompt blood reperfusion is a crucial method to reduce ischaemic stroke injury.^3^, ^4^ However, current clinical treatment methods are extremely limited. There are only two clinically approved methods for treating stroke: (1) mechanical thrombolysis and (2) recombinant tissue plasminogen activator (r‐tPA), which are the only drugs approved by the US Food and Drug Administration. However, due to a limited time window for therapeutic intervention following a stroke, and clinical contraindications in some patients, only 11% of patients are eligible for r‐tPA treatment, and half of these patients do not show clinical improvement.^3^, ^4^ In some cases, restoration of blood supply may cause ischaemia‐reperfusion (I/R) injury to ischaemic brain tissue, which further exacerbates irreversible damage to the brain.^5^, ^6^, ^7^ For instance, extensive damage and death of neurons may cause aggravation of brain tissue damage after I/R. Therefore, in‐depth exploration of the pathogenesis of cerebral I/R injury and the search for effective therapeutic targets remain important unmet needs in the treatment of ischaemic stroke.

Recent studies have shown that protein ubiquitination levels are significantly elevated in neurons after cerebral I/R injury.^8^, ^9^ In turn, I/R injury induces damage to the ubiquitin‐proteasome degradation pathway, which leads to neuronal injury.^10^ Protein ubiquitination and deubiquitination are important post‐translational modifications. Ubiquitination modification is the process by which ubiquitin ligases regulate the stability or activity of proteins by labelling them with ubiquitin chains, resulting in the degradation of proteins. In contrast, deubiquitination leads to the removal of polyubiquitin chains on proteins to reduce protein degradation by ubiquitination. Deubiquitination enzymes (DUBs) may reduce neuronal damage induced by cerebral I/R by decreasing protein ubiquitination levels.^11^, ^12^ At present, the molecular mechanism of deubiquitination modification in regulating cerebral I/R injury is still unclear. Therefore, in‐depth exploration of the molecular mechanisms underlying deubiquitination in I/R injury is of great significance in the search for new therapeutic targets in the treatment of ischaemic stroke.

Ovarian tumour domain‐containing protein 3 (OTUD3) is a member of ovarian tumour proteases (OTUs) family of deubiquitination enzymes located in the cytoplasm. To date, limited studies on OTUD3 have focused mainly in tumour biology. There are very few studies on OTUD3 in brain I/R injury. Moreover, we conducted bioinformatics analysis on the OTU family and other deubiquitinating enzymes using data available online, and it was not found that there were obvious positive results in the I/R injury model. Therefore, we focused on the study of OTUD3. Yuan et al. reported that OTUD3 inhibited the occurrence and development of breast cancer by removing PTEN polyubiquitination modification and maintaining the stability of PTEN protein.^13^ In addition, Pu et al. found that OTUD3 inhibited the occurrence of breast cancer by stabilizing p53 and reducing DNA damage caused by ultraviolet radiation.^14^ Interestingly, Du et al. found that OTUD3 could promote cancer cell growth in lung cancer,^15^ and Xie et al. recently showed that OTUD3 promoted the growth and metastasis of liver cancer cells by stabilizing ACTN4.^16^ These results indicate that OTUD3 has a strong effect on promoting cell growth. Herein, we report the results of experiments in animal and cell models of ischaemic stroke evaluating the changes in OTUD3 following cerebral I/R injury. We found that the expression of deubiquitination enzyme OTUD3 was significantly reduced in neurons after cerebral I/R injury in mice. Moreover, OTUD3 overexpression reduced the mortality rate of cortical neurons in an oxygen glucose deprivation mouse model. We also found that knockout of the *OTUD3* gene further exacerbated cerebral I/R injury. Taken together, these results strongly suggest that OTUD3 may affect the survival and death of neurons after cerebral I/R injury. Moreover, recent studies have found that OTUD3 reduces ferroptosis in hippocampal neurons to inhibit I/R injury, which indirectly supports the findings of our research team.^17^ However, the depth of these studies is relatively limited, and the specific mechanisms of action require further in‐depth investigation. Previous studies have demonstrated that ferroptosis in neurons is increased during cerebral I/R injury, and members of the deubiquitinase family play significant roles in regulating ferroptosis, but their effects vary depending on the specific gene and disease context.^18^, ^19^ For example, USP11 promotes ferroptosis in neurons by stabilizing Beclin 1 and enhancing autophagy,^18^ whereas USP35 reduces ferroptosis by interacting with the membrane ferroportin.^19^ Therefore, we have conducted research on the relationship between OTUD3 and ferroptosis.

We screened PLK1, one of the proteins that interacts most closely with OTUD3, through immunoprecipitation and mass spectrometry analysis. Previous studies have shown that PLK1 is closely related to ferroptosis, and PLK1 knockout aggravates ferroptosis.^20^ Dixon et al. showed that ferroptosis is important for the regulation of programmed cell death, mediated by iron ions.^21^ They found that due to the reduced intracellular transport of cysteine, the production of glutathione (GSH) and the activity of glutathione peroxidase 4 (GPX4) were decreased, leading to weakened inhibition of ROS production by GPX4, thereby resulting in the accumulation of lipid peroxides and ultimately causing cellular damage and death.^21^ Other studies have shown that ferroptosis is involved in regulating cerebral I/R injury, and inhibition of ferroptosis can reduce cerebral I/R injury.^22^, ^23^ In the present study, we found that OTUD3 knockout exacerbated ferroptosis induced by I/R. After cerebral I/R injury, the downregulation of the deubiquitination enzyme OTUD3 inhibited the PI3K/AKT signalling pathway by increasing the ubiquitination of PLK1 and reducing PLK1 protein levels, thereby reducing the abundance of GSH in neurons, inhibiting GPX4 activity, promoting reactive oxygen species (ROS) generation and ultimately promoting neuronal ferroptosis. Overall, our results indicate potential therapeutic targets for the treatment and prevention of cerebral I/R injury.

## 2 METHODS

### 2.1 Animals

C57BL/6J male mice weighing 25 + 1.5 g aged 8–10 weeks were placed in a room with a humidity of 40%–70% at 20–25°C, 12 h of light a day and a day–night cycle. The experiment was carried out after a week of routine adaptive feeding. All animals were fed in standardized cages and were given ordinary feed. In this study, a total of 461 C57BL/6 mice were used, including 170 male mice and 291 pregnant females. Additionally, a total of 147 OTUD3 knockout mice were used, including 93 male mice and 54 pregnant females.

### 2.2 Evans blue staining extravasation experiment

Evans blue dye was used to study the breakdown of the blood–brain barrier. After the mice were anaesthetized, the configured Evans blue dye (diluted with normal saline at 2% concentration) was injected into the tail vein at 2 mL/kg of body weight and circulated for 2 h. The mice were then deeply anaesthetized, and  .9% saline was injected through the apex of the heart with a 20 mL disposable syringe until colourless  .9% saline was obtained in the right atrium, after which the brains were dissected and photographed. After cutting the brain tissue, the Evans blue dye was extracted with 2 mL normal saline and 1.5 mL 60% trichloroacetic acid homogenate. Then the supernatant was obtained by centrifugation at 1000 r/min for 5 min. The absorbance of the supernatant with a wavelength of 620 nm was measured by spectrophotometer. The absorbance was converted into the concentration of Evans blue using the standard curve calculation formula, and the content of the extract of Evans blue in brain tissue was expressed as µg/g tissue.

### 2.3 Nissl staining

After 24 h of cerebral I/R injury, mice were deeply anaesthetized, first injected with normal saline through the heart, and then injected with 4% paraformaldehyde to fix the brain tissue. Fresh brain tissue was fixed in 10% neutral formalin solution for 48 h, cut into 8 µm slices, dewaxed conventionally and washed with distilled water. The slices were soaked in Nishi dyeing solution (G1036; Servicebio) for 2–5 min, washed with water, slightly differentiated with  .1% glacial acetic acid, washed with tap water to terminate the reaction, and the slices were baked in the oven to dry. Then use clean xylene transparent 10 min, neutral gum seal, air dry. Microscope observation, photography, image acquisition and analysis.

### 2.4 Drug treatment

PLK1‐specific inhibitor PLK1‐IN‐6 and PI3K‐specific inhibitor PI3K‐IN‐33 were purchased from MedChemExpress. The ferroptosis‐specific inhibitor ferrostatin‐1 was purchased from Santa Cruz Biotechnology, Inc. According to the instructions provided by the manufacturer, PLK1‐IN‐6 was added to the culture medium with DMSO as the solvent to achieve a concentration of 50 nM or PI3K‐IN‐33 was added to the culture medium to achieve a concentration of 10 µM or ferrostatin‐1 was added to the culture medium to achieve a concentration of 1 µM, and then in vitro experiments were conducted. In in vivo experiments, we used DMSO as a solvent to prepare PLK1‐IN‐6 with a concentration of 1 mg/kg and injected it into mice through the ventricle for follow‐up experiments.

### 2.5 Cerebral perfusion analysis

After anaesthetizing the mice, a midline incision was made to expose the skull. Blood perfusion throughout the cerebral cortex was assessed using a laser speckle imager (PeriCam PSI system; Perimed AB). *System settings*: detection distance 10 cm, laser irradiation area 2 cm × 2 cm. The PSI system uses 1386 × 1034 pixels to calculate regional spatial contrast based on a 3 × 3 degree matrix. After measurement, sterile sutures were used to close the skin. The perfusion data were evaluated using a review software (PIMsoft software version 1.2). The average image was calculated from 60 perfusion images. The average value of fixed‐size area of interest (region of interest (ROI), 60 mm^2^, including bilateral cerebral cortex) during the time period of interest (4–6 s) was selected for cerebral perfusion assessment.

### 2.6 Construct OTUD3 knockout mice

*Vector construction*: According to the design scheme, gRNA was designed, constructed and transcribed in vitro, and donor vector was constructed to verify the correctness of the vector sequence through sequencing. *Microinjection*: The CRISPR/Cas9 system and donor vector samples were microinjected into C57BL/6JGpt mouse‐fertilized eggs. The surviving fertilized eggs after injection were transplanted into the pseudopregnant female mice, and then pregnant and gave birth to young. *Identification of F0 generation mice*: The F0 generation pups born from the recipient mice were numbered by tail clipping and toe clipping at 5–7 days, and genomic DNA was extracted for PCR and sequencing identification to confirm the genotype. *Breeding of positive F0 generation mice*: After the positive F0 generation mice became sexually mature, they mated with wild‐type background mice. The tail and toe numbers of F1 generation mice were clipped at 5–7 days after birth, and genomic DNA was extracted for PCR and sequencing identification to confirm the genotype.

### 2.7 Plasmid construction and lentiviral/plasmid transfection of neurons

The primers were synthesized by Wuhan Miaoling Biotechnology Co., Ltd. I‐5™ 2× High‐fidelity Master Mix High assurance polymerase was used for PCR amplification. After PCR amplification of the target gene, 1% agarose gel was used for electrophoresis detection, and DNA marker was used as a reference to determine the fragment size of the target gene. The above correct target bands were cut and recycled into sterile 1.5 mL EP tubes, and the target DNA fragments were recovered according to the instructions of the rapid agarose gel DNA recovery kit, and the purified target gene products were finally obtained. In the sterile  .2 mL EP reaction tube, the enzyme‐cut vector was subjected to homologous recombination reaction according to the manufacturer's instructions (Qijing Biotechnology Co., Ltd.), and the products were connected to transform the receptor cells. Finally, plasmid extraction and sequencing were performed, and the sequencing results matched 100% with the target sequence. Transfection of lentiviruses carrying OTUD3‐short hairpin (sh)RNA (GeneChem Biotechnology Co., Ltd.), pCMV‐OTUD3, pCMV‐PLK1, pCMV‐IRP2, pCMV‐OTUD3^C76A^ or pCMV‐Control (Qijing Biotechnology Co., Ltd.) was performed according to the manufacturer's protocol, and the next experiment was performed 48 h after cell transfection. The cultured neurons were transfected according to the manufacturer's scheme, and the next experiment was conducted after the successful deletion/amplification was verified by western blot.

### 2.8 Immunocoprecipitation

The collected 1 × 10^7^ 293T cells were washed three times with pre‐cooled 1× phosphate buffered saline (PBS) and cleaved in 1 mL of cell lysate. Appropriate amount of protein‐A/G agar–agar beads were taken and pre‐bound at 4°C for 10–30 min. The lysate (including protease inhibitors) was divided into Input, IP and IgG groups according to 1:4:2. Target antibody Flag‐OTUD3 and IgG antibody were added to IP group and IgG group, respectively, and incubated at 4°C for 12–18 h. Rinse three times with the washing buffer. Then the supernatant was obtained by centrifugation after boiling the loading buffer sample for 10 min. The protein samples were coloured by silver staining after PAGE electrophoresis.

### 2.9 The binding region was determined by immunocoprecipitation

*The eukaryotic expression vectors containing PLK1‐truncated body*: PLK1‐truncated body protein 1 (PLK1‐T1), PLK1‐truncated body protein 2 (PLK1‐T2) and PLK1‐truncated body protein 3 (PLK1‐T3) (designed by Beijing Qingke Biotechnology Co., Ltd.) were transfected into 293T cells, respectively, for immunoprecipitation analysis. The structural regions of the interaction between OTUD3 and PLK1 were observed by western blot.

### 2.10 Ubiquitination experiments

HEK293T cells were transfected with pCMV‐OTUD3, pCMV‐OTUD3^C76A^, pCMV‐Control, Flag‐OTUD3 and HA‐Ub (point mutations of the indicated plasmids of ubiquitin chain were introduced through site‐directed mutagenesis) (Qijing Biotechnology Co., Ltd.), the proteasome inhibitor MG132 was added into the medium and the supernatant was added with PLK1 antibody and mixed overnight. Protein A/G–agarose mixture was added to the supernatant overnight, centrifuged, Tris‐NaCl‐EDTA (TNE) cell lysis solution was added and 2*SDS‐PAGE buffer was added and boiled for 10 min. Finally, western blot analysis was performed to detect the ubiquitination expression of PLK1.

### 2.11 ROS tissue section staining

*Quenched tissue autofluorescence*: Frozen sections of brain tissue were rewarmed at room temperature and moisture was controlled. Draw circles around the tissue with a histochemical pen, add a self‐fluorescence quencher for 5 min and rinse with water for 10 min. Add ROS dye solution (D7008, Sigma‐Aldrich (Shanghai) Trading Co., Ltd.) in the ring and incubate at 37°C for 30 min. The slides were placed in PBS (pH 7.4) and washed by shaking on the decolorizing shaker for three times, 5 min each time. Add the DAPI dye solution (G1012; Wuhan Servicebio Biotechnology Co., Ltd.) and incubate at room temperature in the dark for 10 min. The slides were placed in PBS (pH 7.4) and washed by shaking on the decolorizing shaker for three times, 5 min each time. Anti‐fluorescence quenching sealing tablets. The final image collection: DAPI excitation wavelength 330–380 nm; The excitation wavelength of CY3 is 510–560 nm. DAPI nuclei are blue and ROS positive are red.

### 2.12 GSH content detection

Total GSH levels in the mouse brain were measured according to manufacturer's instructions. According to the operating instructions of the GSH content detection kit (BC1175; Beijing Solarbio Technology Co., Ltd.), the collected samples were fully mixed with the reaction reagents, and then the absorbance at 412 nm of each group was measured with the enzyme label instrument, and finally the GSH content of each group was calculated with the calculation formula.

## 3 5‐HETE ELISA

The experiments were performed using an ELISA kit (No. CED739Ge; Wuhan Uscn Life Science Technology Co., Ltd.) following the manufacturer's detailed experimental protocol. Briefly, samples and standards were prepared and added to the pre‐coated plate, followed by the addition of specific reagents as outlined in the instructions. After incubation and washing steps to remove unbound substances, the substrate solution was added to develop the colour reaction. The reaction was then stopped, and the absorbance of each well was measured at the appropriate wavelength using a microplate reader. Finally, the concentration of 5‐HETE in the samples was calculated by comparing the absorbance values to the standard curve generated from the provided standards, using the formula specified by the manufacturer. This method ensures accurate and reliable quantification of 5‐HETE levels in the samples.

## 4 12‐HETE ELISA

The 12‐HETE ELISA kit (ab133034; Abcam) was used to quantify 12‐HETE levels in the samples, following the detailed experimental protocol provided by the manufacturer. Briefly, samples and standards were prepared and added to the pre‐coated plate. Specific reagents, including detection antibodies and enzyme conjugates, were added sequentially according to the instructions. After incubation and thorough washing steps to remove unbound components, a substrate solution was added to initiate the colour development reaction. The reaction was stopped after the appropriate incubation period, and the absorbance of each well was measured at the specified wavelength using a microplate reader. The concentration of 12‐HETE in the samples was then determined by plotting the absorbance values against the standard curve generated from the provided standards and applying the calculation formula specified by the manufacturer. This method ensures precise and reproducible quantification of 12‐HETE levels in the samples.

## 5 15‐HETE ELISA

The 15‐HETE ELISA kit (ab133035; Abcam) was employed to measure 15‐HETE levels in the samples, adhering to the detailed experimental protocol outlined by the manufacturer. In brief, samples and standards were prepared and added to the pre‐coated plate. Specific reagents, including detection antibodies and enzyme conjugates, were sequentially introduced as per the kit instructions. Following incubation and a series of washing steps to eliminate unbound substances, a substrate solution was added to initiate the colorimetric reaction. The reaction was halted after the designated incubation period, and the absorbance of each well was measured at the specified wavelength using a microplate reader. The concentration of 15‐HETE in the samples was subsequently determined by correlating the absorbance values with the standard curve generated from the provided standards and applying the calculation formula specified by the manufacturer. This approach ensures accurate and reliable quantification of 15‐HETE levels in the samples.

### 5.1 Transmission electron microscopy

After 24 h of MCAO/R, the mice were deeply anaesthetized, perfused with normal saline through the heart and finally perfused with 3% glutaraldehyde for fixation. The brains were immediately removed, and the penumbra of the cerebral cortex tissue was cut into 1 mm × 1 mm × 1 mm pieces and stored in electron microscope fixative (G1102; Servicebio). The cells were rinsed three times with  .1 phosphate buffer PB (pH 7.4) for 15 min each time, and then fixed with 1% osmic acid prepared with  .1 phosphate buffer PB (pH 7.4) in the dark at room temperature for 2 h, and then added 30%–50%–70%–80%–95%–100% sequentially ascending dehydration with 100% alcohol for 20 min each and 100% acetone twice for 15 min each. They were embedded in acetone and 812 embedding agent (90529‐77‐4; SPI) and then placed in an oven at 60°C for polymerization for 48 h. The ultrathin sections at 60–80 nm were stained with 2% uranyl acetate saturated alcohol solution and 2.6% lead citrate solution on an ultra‐thin microtome (Leica UC7; Leica). The images were observed under a transmission electron microscope (HT7800; Hitachi) and analysed.

Under transmission electron microscope, the mitochondrial structure of normal neurons was bilayer membrane structure, and the mitochondrial cristae membrane structure was clear. Neuron ferroptosis occurs when mitochondrial membranes rupture and mitochondrial cristae membranes decrease or disappear.

### 5.2 Local cerebral ischaemia

In this study, thread embolism technique was used to induce transient cerebral ischaemia.^24^ C57BL/6J male mice were randomly selected from each group, and male mice with the same body weight range were selected for the experiments. After treatment with 4% isoflurane (30% O_2_ and 70% N_2_O), a midline neck incision was made, the right external carotid artery (ECA) was exposed and carefully dissected, and the right internal carotid artery (ICA) was occluded (ECA insertion line approximately 22 mm to the right internal carotid artery). Ninety minutes after occlusion, embolic threads were removed and reperfusion was allowed. The ECA was then ligated and the wound was sutured. Body temperature was maintained at 37.0 ± .5°C using a heating pad and heat lamp. At 24 h after MCAO/R, mice were anaesthetized by inhalation of 4% isoflurane and 70% N_2_O and 30% O_2_, and then were perfused with  .9% normal saline. Subsequently, brains were immediately removed and stored in a refrigerator.

### 5.3 Intraventricular injection (i.c.v.)

Mouse lateral ventricles were first located anatomically (2 mm posterior to the anterior fontanelle, 1.5 mm lateral to the sagittal suture and 2.5 mm downward to the skull surface) using a 23‐gauge needle attached to a Hamilton microsyringe through a polyethylene tube. Needle placement in the ventricle was confirmed after removal of several microliters of clear cerebrospinal fluid (CSF) with a microsyringe. After 90 min of middle cerebral artery occlusion per reperfusion, the DMSO‐prepared PLK1‐IN‐6 mixture or DMSO was injected into the lateral ventricles of mice at a rate of 1.0 µL/min.

### 5.4 2,3,5‐Triphenyltetrazolium chloride staining

Mice were sacrificed and brain tissues were removed, followed by 2,3,5‐triphenyltetrazolium chloride (TTC) staining, and the volume of cerebral infarction was examined. Brains were placed on a cooled base and cut into 2 mm coronal slices. Each slice was placed in a 10 cm culture dish, and incubated with a mixture of phosphate buffer and 2% TTC in an oven at 37°C for 30 min. After incubation, the slices were fixed in 4% paraformaldehyde and stored overnight in a refrigerator at 4°C. Images were scanned and analysed using ImageJ software. All images were acquired, processed and analysed using a blind method. The ratio of brain infarction area was calculated using the following formula: infarct area of each group/total brain slice area.^25^

### 5.5 Mice primary cortical neuron culture and OGD/R insult

Cortical neuron cultures were prepared from C57BL/6J mice at 17 days of pregnancy.^5^ Pregnant mice were anaesthetized with 4% isoflurane, 30% O_2_ and 70% N_2_O, and killed by cervical dislocation. After whole‐body disinfection of mice with 70% ethanol, embryos were removed and placed in meningeal culture solution (neural basal culture solution supplemented with 2% B‐27,  .5% foetal bovine serum,  .5 mM l‐glutamate and 25 mM glutamate). The embryonic brain was rapidly fragmented, and cortical brain tissue was harvested and stored at low temperature. Mouse cortical neurons were inoculated into a culture dish coated with poly‐d‐lysine and suspended in the culture medium. Every 3 days, half of the culture medium in the dish was taken out and replaced with maintenance medium (neural basal medium, 2% B‐27 supplement and  .5 mM l‐glutamine). Neurons were cultured for 12 days and used for further experiments.

For OGD/R injury, nerve cells were transferred to a hypoxic, glucose‐free extracellular solution containing 116 mmol/L NaCl,  .8 mmol/L MgSO_4_, 1.8 mmol/L CaCl_2_, 1.0 mmol/L NaH_2_PO_4_, 5.4 mmol/L KCl and 26 mmol/L NaHCO_3_, and cultured in a dedicated humidification chamber at 37°C, 85% N_2_, 5% CO_2_ and 10% H_2_ for 60 min. During the recovery process, fresh maintenance medium containing appropriate concentrations of reagents was introduced to replace the old medium. Cells were cultivated in an incubator at 5% CO_2_ and 95% O_2_ for 24 h.

### 5.6 Western blot

Western blot was performed as previously described.^26^ Polyvinylidene difluoride membranes (Millipore) were incubated with primary antibodies overnight at 4°C. (The primary antibodies used in this experiment included: OTUD3 rabbit polyclonal antibody, 1:1000, cat. no. SAB1306783; Sigma Biotechnology; PLK1 rabbit polyclonal antibody, 1:1000, cat. no. ab109777; Abcam Biotechnology; GPX4 mouse monoclonal antibody, 1:1000, cat. no. sc‐166570; Santa Cruz Biotechnology; AKT mouse monoclonal antibody, 1:1000, cat. no. sc‐81434; Santa Cruz Biotechnology; Phospho‐AKT mouse monoclonal antibody, 1:1000, cat. no. sc‐514032; Santa Cruz Biotechnology; PI3K rabbit polyclonal antibody, 1:1000, cat. no. 4292; Cell Signaling Technology; Phospho‐PI3K rabbit monoclonal antibody, 1:1000, cat. no. 17366; Cell Signaling Technology; β‐actin mouse monoclonal antibody, 1: 3000, cat. no. sc‐8432; Santa Cruz Biotechnology.) The membrane was then labelled with horseradish peroxidase conjugated secondary antibody, and the protein bands were imaged with SuperSignal West Femto Maximum Sensitivity Substrate (Pierce). Blot images were obtained directly from polyvinylidene difluoride membranes using an EC3 imaging system (UVP). Group assignment during the experiment was blinded to the experimenter. The data obtained by western blot were quantified using ImageJ software.

### 5.7 Immunohistochemistry

Isoflurane was overinjected into the hearts of mice. About  .9% saline was infused first, then 4% paraformaldehyde was infused for 24 h at 4°C and finally 30% sucrose and  .1 mol/L phosphate buffer were infused for 72 h. After all the above steps were completed, brain tissues were collected, stored in 4% paraformaldehyde solution overnight at 4°C and cut into 16‐µm coronal sections with the use of a Leica VT1000S vibroknife (Leica Micro‐systems AG). The brain sections were incubated with primary antibodies, rabbit anti‐OTUD3 (1:100) (Proteintech Group, Inc.), mouse anti‐GFAP (astrocyte‐specific protein) (Cell Signaling Technology), mouse anti‐Iba‐1 (microglia‐specific protein) (Cell Signaling Technology) and mouse anti‐NeuN (neuron‐specific nuclear protein) (Abcam), and secondary antibodies (goat anti‐mouse 488 and goat anti‐rabbit 594) were obtained from Eugene Molecular Probes. Experiments were performed blind with the use of an Olympus fluorescence microscope (IX51; Olympus). Analysis was performed using ImageJ software (ImageJ).

### 5.8 Lactate dehydrogenase release and cell viability analyses

Lactate dehydrogenase (LDH) release was measured using the colorimetric CytoTox 96 cytotoxicity kit (J2380; Promag (Beijing) Biotechnology Co., Ltd.). In neuron culture, cell viability was measured according to the ability of neurons to absorb a minimum amount of methyl thiazolyl tetrazolium (MTT) (G4000; Promag (Beijing) Biotechnology Co., Ltd.). Both tests were performed according to the instructions in the manufacturer's protocol.

### 5.9 Neurobehavioural tests

Modified neurological severity score (mNSS) was measured as previously described.^27^ These scores were used for a series of motor, sensory, reflex and balance tests, similar to the contralateral neglect test in humans, and were used to rate neurological function from 0 to 18 (normal score, 0; maximum deficit score, 18).

The beam walking test was used to measure complex neural motor function.^28^ Animals were timed while walking on a 100 cm × 2 cm crossbeam. A box was placed at one end of the beam to provide a sense of security for the animals, and tools were used to generate loud noise, prompting the animals to move towards and enter the box. Researchers rated animals based on the time at which they entered the box – the higher the score, the more severe the neurological deficit.

The adhesive removal test (MST) was used to evaluate forelimb function.^29^ A 3.0 cm × 1.0 cm yellow paper tape was wrapped around the front paw. The typical response of the mice was to try to force out the sleeve by pulling the tape with their mouths or brushing the tape with the opposite paw. The wrapped mice were placed in a cage and observed for 30 s. The first timer was set to run continuously, and the second timer started when the animal attempted to remove the sleeve. The left (diseased)/right (healthy) forelimb functional ratio was recorded, and the test was repeated three times a day, taking the average of the two best scores. Lower ratios indicated more severe neurological deficits.

### 5.10 KEGG enrichment analysis and gene set enrichment analysis

To identify signalling pathways associated with experimental conditions, we performed KEGG enrichment analysis and gene set enrichment analysis (GSEA). Firstly, by comparing gene expression data under different experimental conditions, we screened for significantly differentially expressed genes (DEGs) (*p* < .05, |log2FC| > 1). Subsequently, the DEGs were annotated using the KEGG database, and the enrichment of these genes in specific KEGG pathways was assessed using the gene function annotation analysis tool Metascape (https://metascape.org/gp/index.html#/main/step1). Meanwhile, we employed the GSEA method to assess the enrichment of predefined gene sets (from the MSigDB database) in the gene expression profiles. In the GSEA analysis, the significance of gene sets was evaluated using permutation tests (false discovery rate (FDR) < 0.25). Finally, significantly enriched KEGG pathways and gene sets were visualized using bar plots, enrichment plots and other graphical representations.

### 5.11 Single‐cell sequencing

In this study, we obtained single‐cell RNA‐seq datasets (GSE174574, GSE197731 and GSE227651) from the public database GEO (https://www.ncbi.nlm.nih.gov/geo/) and analysed them using the Seurat software package. Firstly, low‐quality cells and genes were filtered through a data quality control (QC) step, followed by normalization of the data and identification of highly variable genes. Next, dimensionality reduction was performed using principal component analysis, and cell clustering analysis was conducted using the Louvain clustering algorithm. To further reveal the characteristics of cell subpopulations, differential gene analysis was performed based on the clustering results, and the distribution of cells was visualized using UMAP. Finally, the biological functions of the cell subpopulations were interpreted in depth by combining functional annotation of the DEGs and literature information.

### 5.12 Statistical analysis

The data and statistical analysis of this study were performed in accordance with the experimental design and analysis recommendations.^30^ The significance criterion (alpha) was set at  .05. Bonferroni tests were used for appropriate post hoc comparisons, whereas ANOVA was used to assess the statistical significance of differences in data between groups, where applicable. *p* < .05 was considered significant. All results are presented as mean ± SEM.

## 6 RESULTS

### 6.1 Reduced OTUD3 expression in neurons after cerebral I/R injury

**FIGURE 1:**
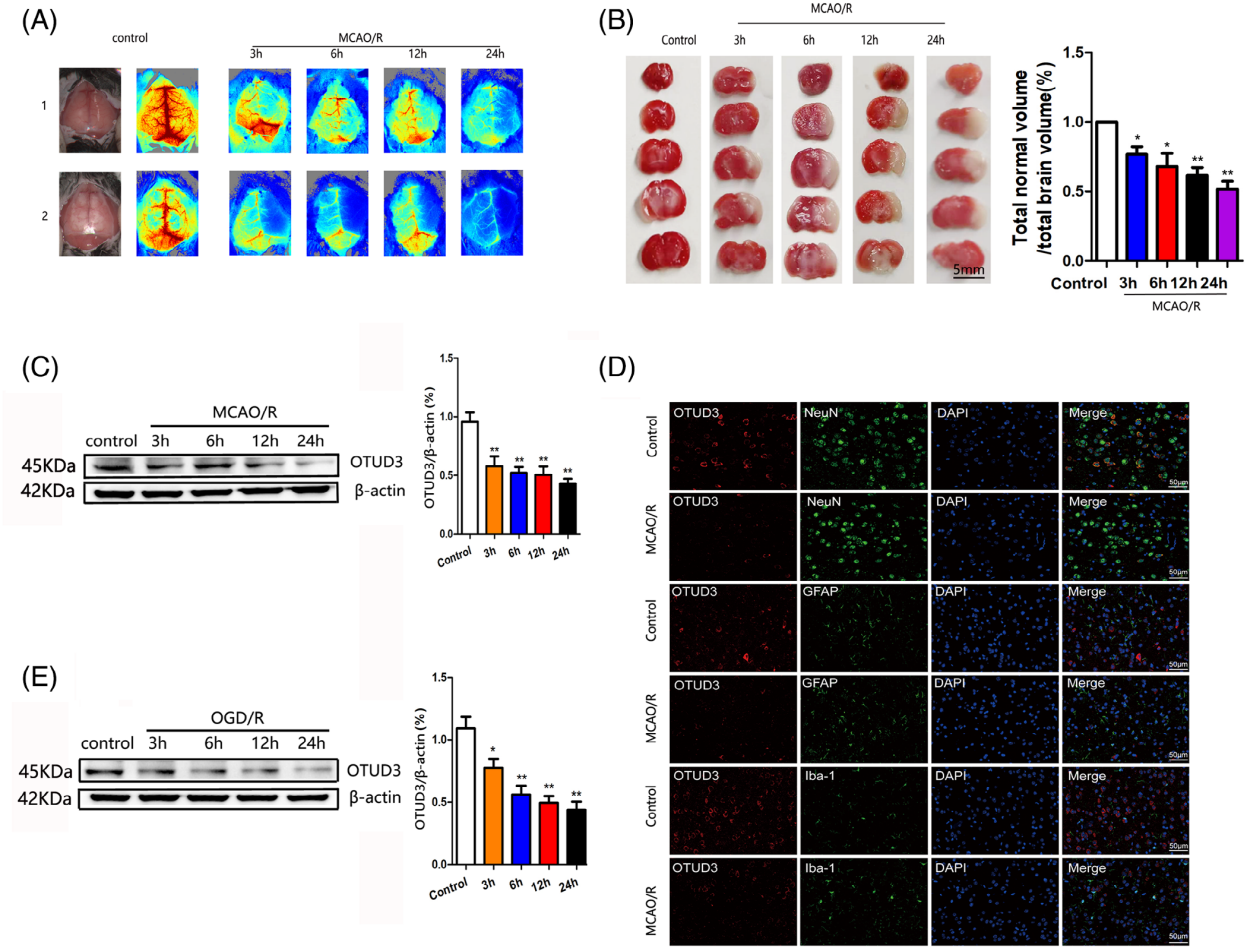
OTUD3 protein expression was decreased after cerebral ischaemia‐reperfusion (I/R) injury. (A) Focal cerebral blood flow was measured by laser Doppler flowmetry. (B) TTC staining was used to show cerebral infarct volume in each group (*n* = 6 for each group; *p < .05 compared to Control; **p < .01 compared to Control; the one‐way ANOVA test, followed by the Bonferroni post hoc test). (C) Western blot was used to detect the expression of OTUD3 protein in each group (*n* = 6 for each group; **p < .01 compared to Control). (D) Double immunofluorescence experiments showed that NeuN, GFAP, Iba‐1 and OTUD3 antibodies were double stained on mouse brain sections after MCAO/R (90 min/24 h). Nuclei were stained with 4ʹ,6‐diamino‐2‐phenylindole (DAPI) (*n* = 6; Scale bar, 50 µm). (E) Western blot was used to detect the expression of OTUD3 protein in each group (*n* = 6 for each group; *p < .05 or **p < .01 compared to Control). Control: mice normal brain tissues and normal mice cortical neurons; MCAO/R 3, 6, 12, 24 h: mice middle cerebral artery occlusion 90 min/reperfusion 3, 6, 12, 24 h. OGD/R 3, 6, 12, 24 h: neurons transferred to a deoxygenated, glucose‐free extracellular solution for 90 min/restore oxygen and glucose 3, 6, 12, 24 h.

To investigate whether OTUD3 was involved in cerebral I/R injury, we first detected the expression of OTUD3 in the cortical brain tissue of mice after cerebral I/R injury. We constructed a mouse MCAO/R injury model and measured focal cerebral blood flow by laser Doppler flowmetry. Figure 1A shows that blood flow in the left hemisphere of mice in the MCAO/R group was significantly reduced, and the blood flow in the left hemisphere of mice decreased more obviously as the duration of reperfusion injury went on. TTC staining experiments showed that the left brain tissue of the mice in the MCAO/R group was obviously infarcted. As the reperfusion injury time was prolonged, the volume of cerebral infarction gradually increased (Figure 1B). Western blot experiments showed that the expression of OTUD3 protein in the penumbra on the side of I/R injury was significantly downregulated (Figure 1C). Microglial cell marker Iba‐1, astroglial cell marker GFAP and neuron marker NeuN and OTUD3 were, respectively, used for immunofluorescence staining. The results indicated that OTUD3 was decreased in each of these cells after 24 h induction by MCAO (Figure 1D). The same results were observed in in vitro experiments, where the OGD/R injury model was constructed in primary cultured mouse cortical neurons, and OTUD3 protein expression was significantly decreased in OGD/R‐injured neurons compared with the control group (Figure 1E). In vitro, we also detected the neuronal survival rate at different time points after OGD/R injury. The results showed that the neuronal survival rate gradually decreased with the reperfusion time after OGD/R injury. The experimental results demonstrated the success of the OGD/R injury model and the reliability of the experimental conclusions (Figure S1A). These results suggest that OTUD3 expression is reduced in neurons after cerebral I/R injury, suggesting that OTUD3 may be involved in the pathogenesis of cerebral I/R injury.

### 6.2 OTUD3 regulates brain injury after I/R

**FIGURE 2:**
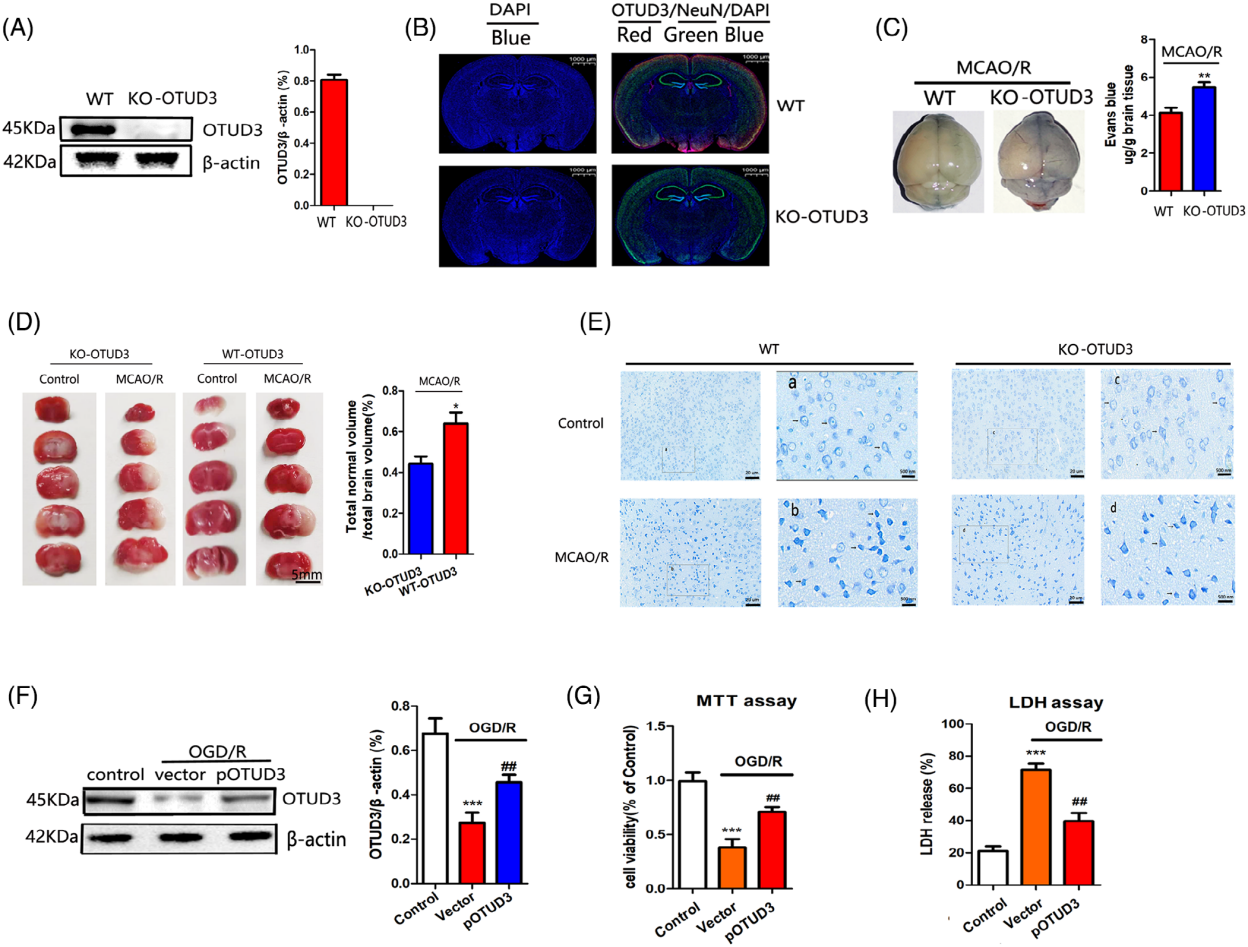
OTUD3 regulates brain injury after ischaemia‐reperfusion (I/R). (A) Western blot assay showed the expression of OTUD3 protein in different groups. (B) Immunofluorescence assay showed the expression of OTUD3 in different groups (*n* = 6; Scale bar, 1000 µm). (C) The Evans blue leakage test showed the level of Evans blue dye leakage in each group (*n* = 6 in each group;**p < .01 compared to WT; the one‐way ANOVA test, followed by the Bonferroni post hoc test). (D) TTC staining was used to show the cerebral infarct volume in each group (*n* = 6 in each group;*p < .05 compared to WT). (E) Nissl staining was used to evaluate the morphological changes of neurons in each group caused by OTUD3 deficiency (*n* = 6; Scale bar, 20 µm). (F) Western blot was used to detect the expression of OTUD3 protein in each group (*n* = 6 in each group;***p < .001 compared to Control; ^##^ *p* < .01 compared to Vector). (G) MTT assay showed the survival rate of neurons in each group (*n* = 6 in each group;***p < .001 compared to Control; ^##^ *p* < .01 compared to Vector). (H) Lactate dehydrogenase (LDH) assay showed the release level of cytotoxic LDH in each group (*n* = 6 in each group; ***p < .001 compared to Control; ^##^ *p* < .01 compared to Vector). WT: OTUD3 wild‐type mice. KO‐OTUD3: OTUD3 knockout mice. Control: mice normal brain tissues and normal mice cortical neurons; MCAO/R: mice middle cerebral artery occlusion 90 min/reperfusion 24 h. OGD/R: neurons transferred to a deoxygenated, glucose‐free extracellular solution for 90 min/restore oxygen and glucose. OGD/R +Vector: The neurons were transfected with empty vector plasmid and OGD/R model was established. OGD/R +pOTUD3: The neurons were transfected with pMCV‐OTUD3 full‐length plasmid to establish the OGD/R model.

Next, we used OTUD3 knockout mice to explore the role of OTUD3 in cerebral I/R injury. Figure S1B shows the overall strategy of OTUD3 gene knockout mice. Figure S1C shows the PCR primers that were used, and Figure S1D shows that the *OTUD3* gene was successfully knocked out in mice numbers 4–5, 7–11, 17 and 22. Western blot analysis showed that OTUD3 protein was not expressed in OTUD3 knockout mice (Figure 2A), and immunofluorescence experiments also showed that OTUD3 protein was not expressed in the neurons of OTUD3 knockout mice (Figure 2B). An MCAO/R injury model was constructed in wild‐type and OTUD3 knockout mice. Evans blue staining extravasation experiment showed that OTUD3 knockout exacerbated the leakage of I/R‐induced blood–brain barrier 24 h after MCAO reperfusion injury (Figure 2C). TTC staining experiments showed that the infarct volume of OTUD3 knockout mice was significantly higher than wild‐type mice (Figure 2D). We also assessed the effect of OTUD3 deficiency on neuronal morphological changes induced by I/R injury by Nissl's staining. As shown in Figure 2E, cells in the cortex and striatum of the control side of wild‐type and OTUD3 knockout mice were evenly stained, with larger cells and abundant cytoplasmic compartments. Neurons on the I/R side were sparsely distributed and their cell bodies were atrophied. In OTUD3 knockout mice, cell body atrophy was more pronounced and neuronal damage was more severe. These data indicate that OTUD3 deletion exacerbated I/R‐induced brain injury. To further verify these findings, we constructed an OGD/R injury model in mouse cortical neurons and overexpressed OTUD3 (Figure 2F). MTT and LDH experiments showed that overexpression of OTUD3 increased the survival rate of neurons and reduced the release of cytotoxic substance LDH after I/R (Figure 2G,H). The above results indicate that changes in OTUD3 expression are involved in regulating cerebral I/R injury.

### 6.3 Knockout of OTUD3 increases ferroptosis in the brain after I/R

**FIGURE 3:**
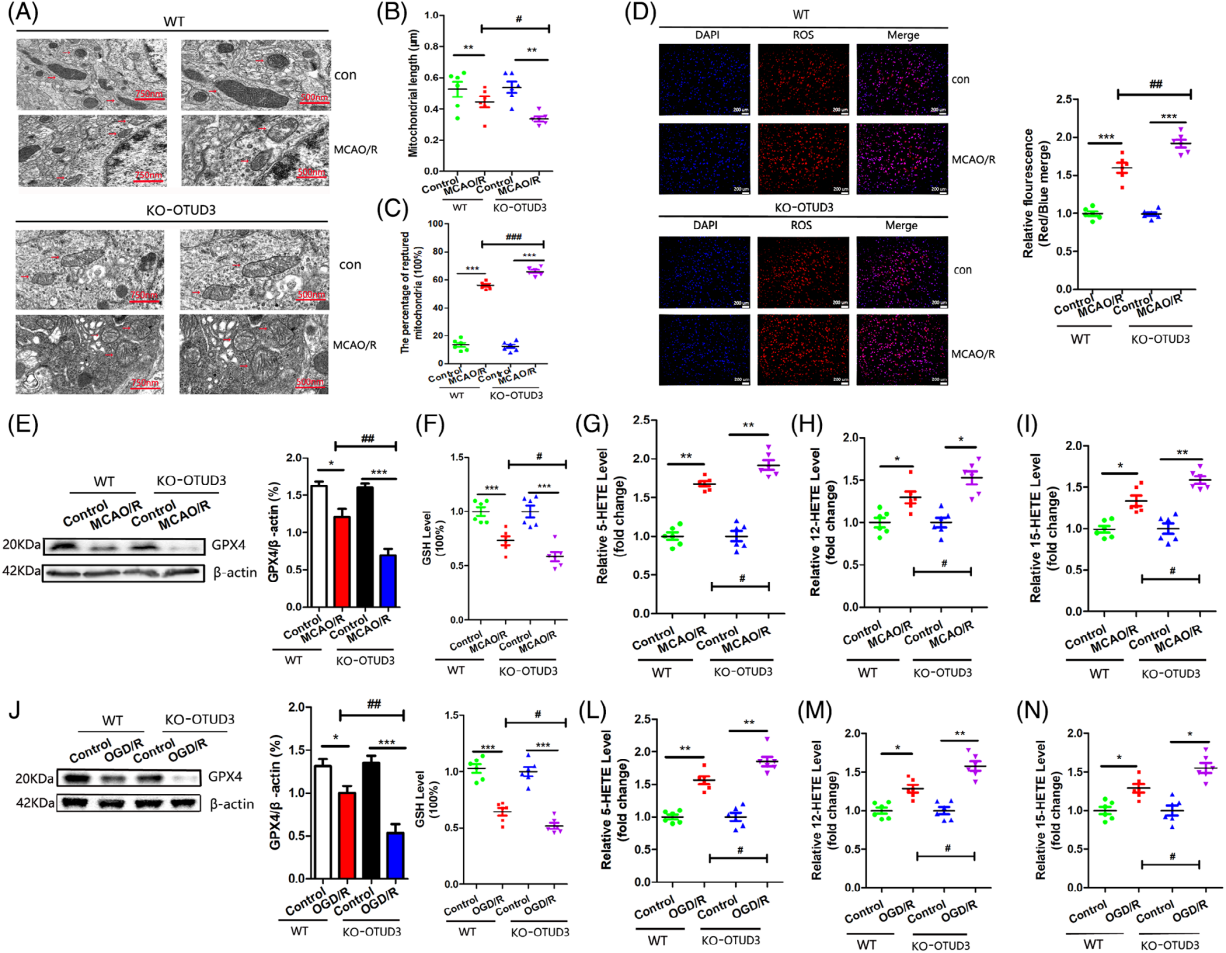
Knockout of OTUD3 increases ferroptosis in the brain after ischaemia‐reperfusion (I/R). (A) Typical pictures of mitochondrial morphological changes in each group were observed by transmission electron microscope, and the red arrows indicated mitochondria (scale bars, 750 and 500 nm). (B) The mitochondrial length was measured by transmission electron microscopy and analysed statistically (*n* = 6; **p < .01 compared to Control; ^#^ *p* < .05 compared to WT MCAO/R; the one‐way ANOVA test, followed by the Bonferroni post hoc test). (C) Transmission electron microscopy was used to observe and measure the percentage of ruptured mitochondria in each group, and the average value was taken for statistical analysis (*n* = 6; ***p < .001 compared to Control; ^###^ *p* < .001 compared to WT MCAO/R). (D) Immunofluorescence was used to detect the changes of ROS in each group and statistical analysis was performed (*n* = 6; Red: ROS, blue: DAPI. Scale bar, 200 µm; ***p < .001 compared to Control; ^##^ *p* < .01 compared to WT MCAO/R). (E) Western blot was used to detect the changes of GPX4 protein in each group (*n* = 6; *p < .05 or ***p < .001 compared to Control; ^##^ *p* < .01 compared to WT MCAO/R). (F) The GSH content of each group was detected by GSH content detection kit and statistically analysed (*n* = 6; ***p < .001 compared to Control; ^#^ *p* < .05 compared to WT MCAO/R). (G–I) 5‐HETE, 12‐HETE and 15‐HETE kits were used to detect the levels of 5‐HETE, 12‐HETE and 15‐HETE in each group (*n* = 6; *p < .05 or **p < .01 compared to Control; ^#^ *p* < .05 compared to WT MCAO/R). (J) Western blot was used to detect the changes of GPX4 protein in each group (*n* = 6; *p < .05 or ***p < .001 compared to Control; ^##^ *p* < .01 compared to WT OGD/R). (K) The GSH content of each group was detected by GSH content detection kit and statistically analysed (*n* = 6; ***p < .001 compared to Control; ^#^ *p* < .05 compared to WT OGD/R). (L–N) 5‐HETE, 12‐HETE and 15‐HETE kits were used to detect the levels of 5‐HETE, 12‐HETE and 15‐HETE in each group (*n* = 6; *p < .05 or **p < .01 compared to Control; ^#^ *p* < .05 compared to WT OGD/R). WT: OTUD3 wild‐type mice. KO‐OTUD3: OTUD3 knockout mice. Control: mice normal brain tissues and normal mice cortical neurons. MCAO/R: mice middle cerebral artery occlusion 90 min/reperfusion 24 h. OGD/R: neurons transferred to a deoxygenated, glucose‐free extracellular solution for 90 min/restore oxygen and glucose.

We examined the effect of OTUD3 deletion on ferroptosis following I/R and found that OTUD3 knockout aggravates I/R‐induced mitochondrial damage. Compared with the control group, mitochondrial volume was smaller, mitochondrial length was reduced, mitochondrial membrane rupture was increased in the brain tissues of mice in the MCAO/R group and mitochondrial damage was further aggravated after OTUD3 knockout (Figure 3A–C). These morphological characteristics are the typical changes associated with cell ferroptosis, suggesting that the knockout of OTUD3 promotes ferroptosis in I/R. Compared with the control group, the generation of ROS in the brain tissue of mice in the MCAO/R group increased, and knockout of OTUD3 promoted a further increase in ROS in the brain tissue of MCAO/R mice (Figure 3D). Levels of both GSH and GPX4 declined 24 h after cerebral I/R injury in mice, and OTUD3 deficiency exacerbated the blockade of this antioxidant system (Figure 3E,F). The ferroptosis signature products of 5‐HETE, 12‐HETE and 15‐HETE were significantly increased in mouse brains after 24 h of MCAO/R, and OTUD3 deficiency further increased the levels of 5‐HETE, 12‐HETE and 15‐HETE (Figure 3G–I). To further confirm the relationship between OTUD3 and ferroptosis, we constructed an OGD/R model in primary cortical neurons of wild‐type and OTUD3 knockout mice and examined changes in the levels of GSH, GPX4, and the ferroptosis signature products of 5‐HETE, 12‐HETE and 15‐HETE. Consistent with our in vivo findings, OTUD3 deficiency led to a further decrease in OGD/R injury‐mediated neuronal GSH and GPX4 content (Figure 3J,K), as well as a further increase in OGD/R injury‐mediated neuronal levels of 5‐HETE, 12‐HETE and 15‐HETE (Figure 3L–N). To further clarify whether OTUD3 can affect neuronal ferroptosis in I/R injury, we added the ferroptosis‐specific inhibitor ferrostatin‐1 to neurons and detected the levels of the ferroptosis biomarkers 5‐HETE, 12‐HETE and 15‐HETE. The results showed that in OGD/R injury, compared with the OGD/R group of neurons from OTUD3 knockout mice, the addition of ferrostatin‐1 significantly attenuated the increase in 5‐HETE, 12‐HETE and 15‐HETE levels caused by OTUD3 knockout (Figure S1E–G). These results indicate that the addition of the ferroptosis‐specific inhibitor significantly weakened the increase in ferroptosis levels caused by OTUD3 knockout. These results provide morphological and molecular evidence that neuronal ferroptosis occurs in cerebral I/R and that OTUD3 deficiency exacerbates I/R‐induced ferroptosis, indicating that OTUD3 is involved in regulating ferroptosis.

### 6.4 Binding of OTUD3 to PLK1

**FIGURE 4:**
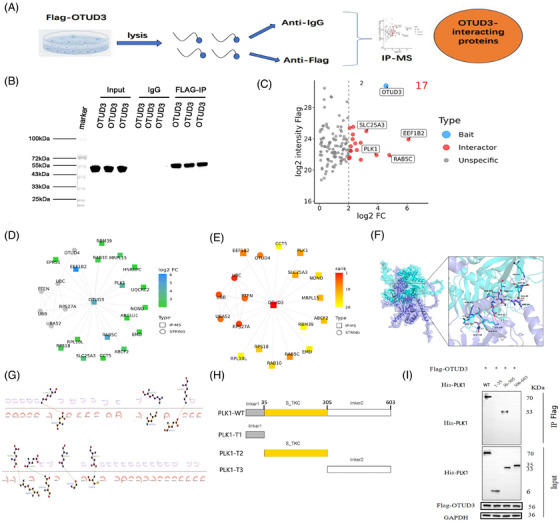
Co‐immunoprecipitation and mass spectrometry were used to detect OTUD3 interacting proteins. (A) Schematic diagram of the experimental line of co‐immunoprecipitation‐mass spectrometry. (B) The results of co‐immunoprecipitation experiment showed that Flag‐OTUD3 enrichment was qualified (*n* = 3). (C) The proteins with >4‐fold difference between Flag‐OTUD3 group and IgG group were preliminarily screened as OTUD3 protein interaction proteins (*n* = 3). (D) The protein interaction network diagram with OTUD3 shown in IP‐MS experiment and STRING database showed that PLK1 was closely related to the bait protein and had obvious differences. (Each node represents an interacting protein, and the line indicates that there is an interaction between the proteins. The central node of the network diagram is the bait protein OTUD3, the circular node is the known interacting proteins of the bait protein retrieved in the STRING database, the square node represents the interacting proteins identified in the IP‐MS experiment and the superposition of the square node and the circular node represents the known interacting proteins identified in the STRING database. Node colours indicate fold differences in protein between experimental and control samples in IP‐MS experiments). (E) Each protein node of the protein interaction network in the previous section was scored based on the CytoHubba importance scoring algorithm MCC, and the top 20 nodes were used to construct the key interaction protein sub‐network. Among them, PLK1 ranked the top in the importance of the screened interaction proteins. (Each node represents an interacting protein, and the line indicates that there is an interaction between the proteins. The central node of the network diagram is the decoy protein, the circular node is the known interacting proteins of the decoy protein retrieved in the STRING database, the square node represents the interacting proteins identified in this IP‐MS experiment, and the diamond node represents the known interacting proteins identified in this experiment in the STRING database. The node colour indicates the rank in the importance score of the corresponding algorithm.) (F, G) The amino acid binding sites of OTUD3 and PLK1 proteins were predicted using molecular docking. (H) A schematic illustration of PLK1 fragments, WT: full‐length wild‐type plasmid, PLK1‐T1: linker1 (1‐35aa), PLK1‐T2: S_TKC (36‐305aa), PLK1‐T3: linker2 (306‐603aa), amino acid residues are indicated by numbers. (I) CO‐IP results showed the interaction between Flag‐OTUD3 and different PLK1 plasmids. After 293T cells were transfected with Flag‐OTUD3 plasmid, the wild‐type His‐PLK1 full‐length plasmid, His‐PLK1‐T1 plasmid, His‐ PLK1‐T2 plasmid and His‐PLK1‐T3 plasmid were transfected, respectively, and then the subsequent IB and IP experiments were performed.

To further explore the downstream molecular mechanism by which OTUD3 regulates ferroptosis in the brain after I/R, we performed co‐immunoprecipitation‐mass spectrometry experiments (Figure 4A). Firstly, co‐immunoprecipitation (Co‐IP) was performed using anti‐Flag‐OTUD3 antibody in 293T cells. The western blot results showed protein bands bound to Flag‐OTUD3 (Figure 4B). The candidate interacting proteins of OTUD3 were analysed by mass spectrometry in 293T cells. Compared with the IgG control group, 17 proteins with a difference >4‐fold in the Flag‐OTUD3 group were initially screened as interacting proteins of OTUD3. The top four proteins were PLK1, SLC25A3, EEF1B2 and RAB5C, in which PLK1 is the protein that is most closely related to OTUD3 (Figure 4C–E and Figure S2). Analyses of peptide length distribution, missed cleavage site distribution, mass deviation analysis and elimination of common contaminating proteins provide supporting evidence for the reliability of our mass spectrometry results (Figure S3). In previous studies, PLK1 was shown to be involved in ferroptosis in tumour cells and played a key role in promoting tumour cell growth.^19^ We predicted the binding of OTUD3 and PLK1 proteins using molecular docking techniques. As shown in Figure 4F,G, there were multiple amino acid binding sites between the two proteins, such as ASN155‐ARG557, GLN153‐GLU555, GLY47‐GLN560, CYS48‐GLU401, ASP42‐LYS66, GLU51‐LYS556 and ARG40‐GLU42. This provides spatial support for their interaction. We next used co‐immunoprecipitation and western blot methods to analyse the specific fragments of OTUD3 and PLK1 that bind to each other. Firstly, we constructed different truncations of PLK1, including PLK1‐truncated protein 1 (PLK1‐T1): linker1 (1‐35aa), PLK1‐truncated protein 2 (PLK1‐T2): S_TKC (36‐305aa) and PLK1‐truncated protein 3 (PLK1‐T3): linker2 (306‐603aa) (Figure 4H and Figure S4). Among them, PLK1‐T2 included the amino acid binding site predicted by molecular docking technology. Cells were transfected with wild‐type PLK1 plasmid, PLK1‐T1 plasmid, PLK1‐T2 plasmid and PLK1‐T3 plasmid. The results showed that PLK1 protein was detected in both the group transfected with wild‐type PLK1 plasmid and the group transfected with PLK1‐T2 plasmid. No PLK1 protein was detected in the groups transfected with PLK1‐T1 and PLK1‐T3 (Figure 4I). These results indicate that OTUD3 interacted with PLK1, and PLK1‐T2 (36–305) is the sequence of PLK1 that binds to OTUD3.

### 6.5 Upregulation of PLK1 expression by OTUD3 reduces cerebral I/R‐induced ferroptosis

**FIGURE 5:**
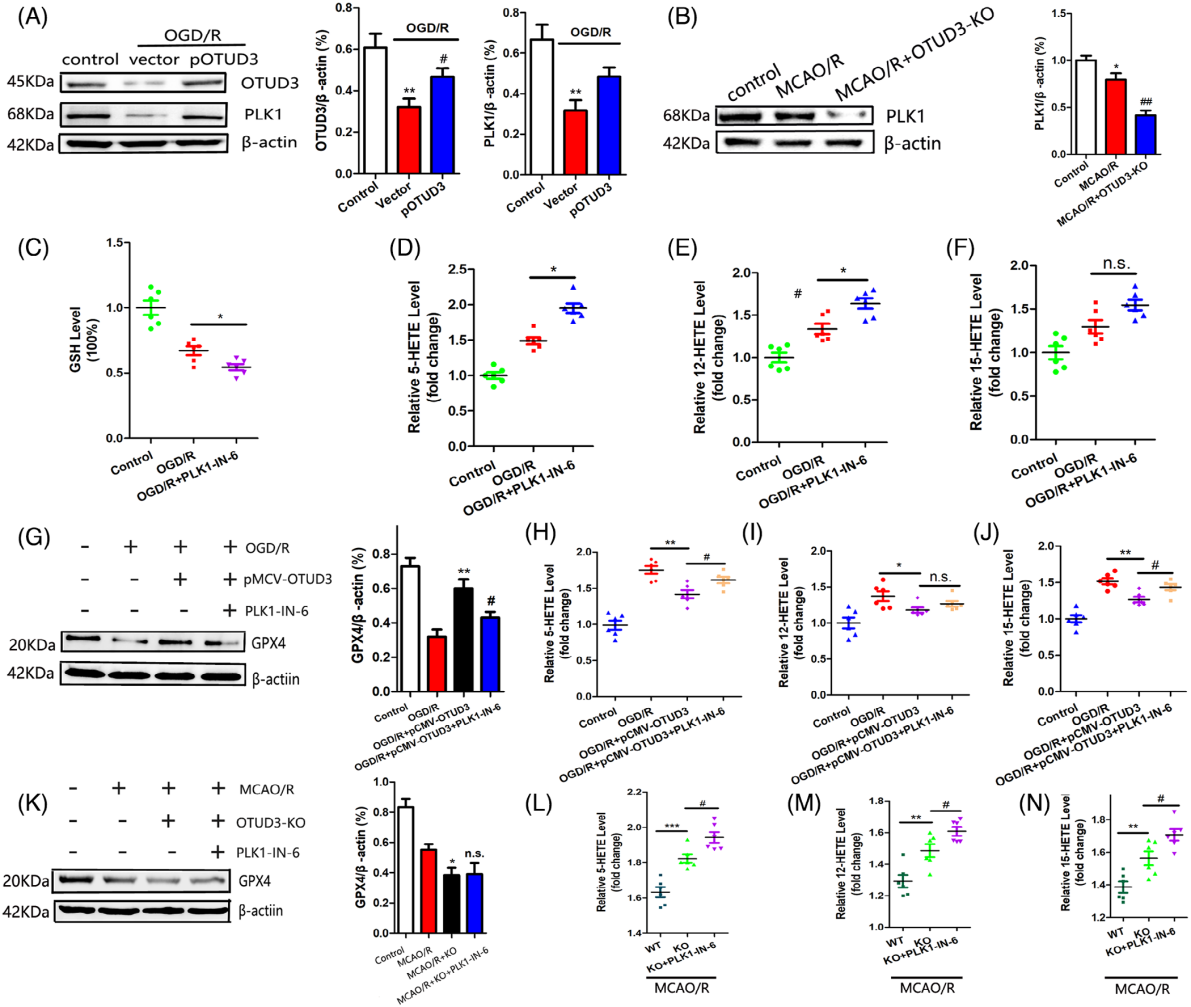
OTUD3 reduces ferroptosis in cerebral ischaemia‐reperfusion (I/R) by upregulating PLK1. (A) Western blot was used to detect the protein expression levels of OTUD3 and PLK1 in each group (*n* = 6; **p < .01 compared to Control; ^#^ *p* < .05 compared to OGD/R + Vector; the two‐way ANOVA test, followed by the Bonferroni post hoc test). (B) In vivo, western blot was used to detect the expression of PLK1 protein in each group (*n* = 6; *p < .05 compared to Control; ^##^ *p* < .01 compared to MCAO/R). (C) The GSH content of each group was detected by GSH content detection kit and statistically analysed. Mouse cortical neurons were treated with OGD/R, and PLK1‐IN‐6, a specific inhibitor of PLK1, was added for subsequent experiments (*n* = 6; *p < .05 compared to OGD/R). (D–F) 5‐HETE, 12‐HETE and 15‐HETE kits were used to detect the levels of 5‐HETE, 12‐HETE and 15‐HETE in each group (*n* = 6; *p < .05 compared to OGD/R; n.s., no significance compared to OGD/R). (G) Western blot was used to detect the expression of GPX4 protein in each group (*n* = 6; **p < .01 compared to OGD/R; ^#^ *p* < .05 compared to OGD/R+pCMV‐OTUD3). (H–J) 5‐HETE, 12‐HETE and 15‐HETE kits were used to detect the levels of 5‐HETE, 12‐HETE and 15‐HETE in each group (*n* = 6; *p < .05 or **p < .01 compared to OGD/R; ^#^ *p* < .05 compared to OGD/R+pCMV‐OTUD3; n.s., no significance compared to OGD/R+pCMV‐OTUD3). (K) Western blot was used to detect the expression of GPX4 protein in each group (*n* = 6; *p < .05 compared to MCAO/R; n.s., no significance compared to MCAO/R+OTUD3‐KO). (L–N) 5‐HETE, 12‐HETE and 15‐HETE kits were used to detect the levels of 5‐HETE, 12‐HETE and 15‐HETE in each group (*n* = 6  ; **p < .01 or ***p < .001 compared to MCAO/R+WT; ^#^ *p* < .05 compared to MCAO/R+OTUD3‐KO). Control: normal mice cortical neurons or OTUD3 wild‐type mice normal brain tissues; OGD/R: neurons transferred to a deoxygenated, glucose‐free extracellular solution for 90 min/restore oxygen and glucose; OGD/R+Vector: neurons were transfected with empty vector plasmid and OGD/R model was established. OGD/R+pOTUD3: the OGD/R model was established after neurons were transfected with OTUD3 plasmid. OGD/R+pOTUD3+PLK1‐IN‐6: after neurons were transfected with OTUD3 plasmid and PLK1 inhibitor PLK1‐IN‐6, the OGD/R model was established. MCAO/R+WT: OTUD3 wild‐type mice middle cerebral artery occlusion 90 min/reperfusion 24 h. MCAO/R+OTUD3‐KO: OTUD3 knockout mice middle cerebral artery occlusion 90 min/reperfusion 24 h. MCAO/R+OTUD3‐KO+PLK1‐IN‐6: the MCAO/R model was established in OTUD3 knockout mice after intraventricular injection of PLK1‐IN‐6.

We performed a validation analysis of PLK1 expression levels in neurons of MCAO mice at the single‐cell level. Firstly, we analysed the 3 h, 12 h, 72 h and sham‐operated brain tissues from the PRJNA912889 mouse MCAO dataset published in PubMed. After quality control, normalization and removal of doublets, the samples contained a total of 37 858 qualified cells. After dimensionality reduction by principal component analysis and UMAP, these cells were identified into clusters and annotated based on the celltypist algorithm. We obtained the distribution of 11 types of cells in brain tissue from mice at different time points after MCAO injury, including neurons, fibroblasts, macrophages, microglia, oligodendrocytes, endothelial cells, parietal cells, astrocytes, B cells, neutrophils and T cells (Figure S5A–D). Subsequently, we analysed PLK1 expression in various cell types. The expression of PLK1 in neurons was significantly reduced at 12 and 72 h after MCAO injury (Figure S5E,F). Similarly, we identified reduced expression of PLK1 protein in the cortical brain tissue in mice with MCAO/R injury and in primary neurons stimulated by OGD/R (Figure 5A,B), which was consistent with the single‐cell sequencing results. We also found that overexpression of OTUD3 could upregulate the expression of PLK1 in neurons cultured in vitro, and knockout of OTUD3 in mice downregulated the expression of PLK1 (Figure 5A,B). We speculated that the reduction of PLK1 might be related to the reduction of OTUD3 induced by cerebral I/R injury. Compared with the OGD/R group, treatment with the PLK1‐specific inhibitor PLK1‐IN‐6 resulted in further reduction of the negative regulator GSH of ferroptosis in mouse cortical neurons induced by OGD/R (Figure 5C) and promoted further production of the ferroptosis signature products of 5‐HETE, 12‐HETE and 15‐HETE (Figure 5D–F). These results indicated that inhibition of PLK1 exacerbated I/R‐induced neuronal ferroptosis. Studies have shown that OTUD3 alleviates ferroptosis in hippocampal neurons by stabilizing IRP2.^17^ Therefore, we explored the relationship between PLK1 and IRP2. Western blot experiments showed that in OGD/R injury, overexpression of PLK1 did not significantly change the protein expression of IRP2, and overexpression of IRP2 also did not significantly change the protein expression of PLK1 (Figure S6A,B). The results suggest that in I/R injury, PLK1 and IRP2 may not have a direct upstream–downstream relationship and might functionally be in a parallel relationship. We performed KEGG analysis on the differential genes related to ferroptosis in the single‐cell RNA‐seq data to assess the enrichment levels of ferroptosis‐related pathways in neurons at different time points (sham, MCAO 3 h, MCAO 12 h and MCAO 3 days). We observed that the ferroptosis‐related pathways were significantly enriched at MCAO 3 h, MCAO 12 h and MCAO 3 days, with the most pronounced enrichment occurring at MCAO 3 h (Figure S6C–E). The results suggest that ferroptosis may play a role in neuronal death during the MCAO injury period. Furthermore, to clarify the importance of ferroptosis in neuronal death‐related pathways following MCAO injury, we obtained gene sets for five cell death modalities – ferroptosis, apoptosis, necroptosis, pyroptosis and autophagy – from the authoritative Reactome database. We then performed KEGG analysis to count the enrichment of genes associated with these five cell death‐related pathways in neurons. The results showed that ferroptosis ranked first among all cell death‐related pathways (Figure S6F). These findings indicate that ferroptosis may be the predominant mode of neuronal death following MCAO injury.

Subsequently, we constructed a neuron OGD/R model. After 24 h of OGD/R, the level GPX4, a negative regulator of ferroptosis, decreased in neurons. Transfection of the OTUD3 full‐length plasmid resulted in a modest increase in GPX4 level (Figure 5G). In addition, the overexpression of OTUD3‐mediated GPX4 production was suppressed following treatment with PLK1‐IN‐6 (Figure 5G). The ferroptosis signature products 5‐HETE, 12‐HETE and 15‐HETE were significantly increased 24 h after neuronal OGD/R. Overexpression of OTUD3 led to decreased production of 5‐HETE, 12‐HETE and 15‐HETE, and PLK1‐IN‐6 drug treatment suppressed OTUD3‐mediated reduction of 5‐HETE, 12‐HETE and 15‐HETE (Figure 5H–J). These results suggested that inhibition of PLK1 suppressed the inhibitory effect of OTUD3 overexpression on ferroptosis. In our in vivo experiment, OTUD3 knockout mice were used to construct an MCAO/R model and PLK1‐IN‐6 was injected intracerebroventricularly. GPX4 levels were not altered in the MCAO/R+OTUD3 knockout group versus the MCAO/R+OTUD3 knockout+PLK1‐IN‐6 group (Figure 5K). However, PLK1‐IN‐6 further promoted the generation of 5‐HETE, 12‐HETE and 15‐HETE mediated by OTUD3 deletion (Figure 5L–N). The results suggested that inhibition of PLK1 further promoted neuronal ferroptosis mediated by OTUD3 deficiency. The above results indicated that PLK1 was involved in regulating the occurrence of neuronal ferroptosis in OTUD3‐mediated cerebral I/R injury.

### 6.6 Activation of the PI3K/AKT signalling pathway by PLK1 reduces neuronal ferroptosis after cerebral I/R injury

**FIGURE 6:**
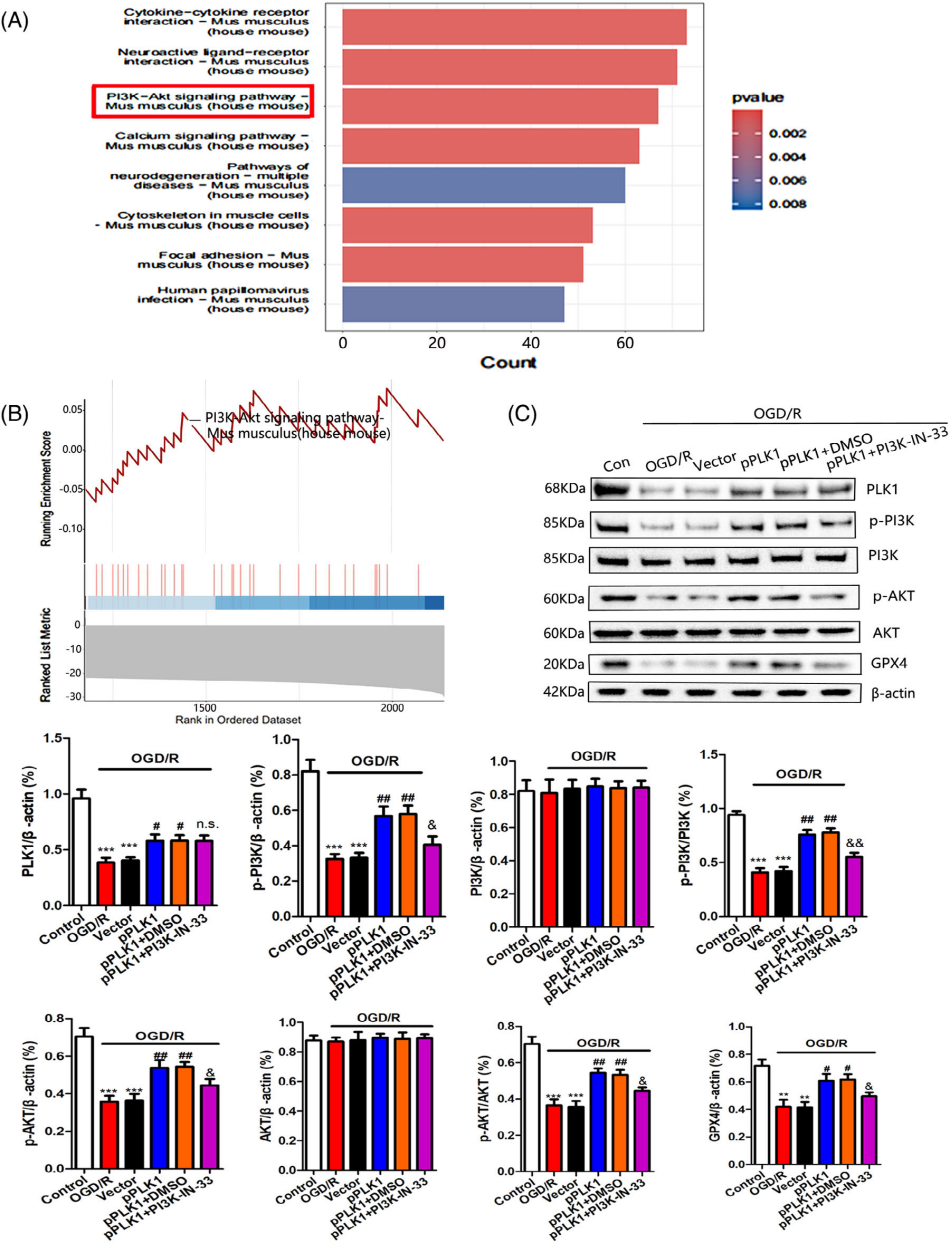
In cerebral ischaemia‐reperfusion (I/R) injury, PLK1 activates the PI3K/AKT signalling pathway to reduce ferroptosis. (A) KEGG pathway enrichment analysis showed that PI3K/AKT signalling pathway was closely related to cerebral I/R injury. (B) GSEA enrichment analysis also showed that PI3K/AKT signalling pathway was significantly inhibited after cerebral I/R injury. Pathway analysis was performed using clusterprofiler 4.10.1. (C) Western blot experiments showed the protein levels of PLK1, p‐PI3K, PI3K, p‐AKT, AKT and GPX4 in each group (*n* = 6 for each group; **p < .01 or ***p < .001compared to Control; ^#^ *p* < .05 or ^##^ *p* < .01 compared to OGD/R + Vector; ^&^ *p* < .05 or ^&&^ *p* < .01 compared to OGD/R + pPLK1+DMSO; n.s., no significance compared to OGD/R + pPLK1+DMSO; the two‐way ANOVA test, followed by the Bonferroni post hoc test). Control: normal mice cortical neurons. OGD/R: neurons transferred to a deoxygenated, glucose‐free extracellular solution for 90 min/restore oxygen and glucose. OGD/R+Vector: the neurons were transfected with empty vector plasmid and OGD/R model was established. OGD/R+pPLK1: the neurons were transfected with PLK1 plasmid to establish the OGD/R model. OGD/R+pPLK1+DMSO: the neurons were transfected with PLK1 plasmid and added with the same volume of DMSO to establish the OGD/R model. OGD/R+pPLK1+ PI3K‐IN‐33: the neurons were transfected with PLK1 plasmid and added with PI3K inhibitor PI3K‐IN‐33 to establish the OGD/R model.

Through KEGG enrichment analysis, we found that the PI3K/AKT signalling pathway was significantly enriched after cerebral I/R injury (Figure 6A). At the same time, GSEA also showed that the PI3K/AKT signalling pathway was significantly inhibited after cerebral I/R injury (Figure 6B). Previous studies have demonstrated that PLK1 can regulate the PI3K/AKT signalling pathway.^31^ Therefore, this study further explores the relationship between PLK1 and the PI3K/AKT signalling pathway. Western blot results showed that upregulation of PLK1 expression in OGD/R‐injured neurons increased the levels of p‐PI3K and p‐AKT proteins. There was no significant change in the total protein levels of PI3K and AKT, and the levels of GPX4 were increased. The inhibition of PI3K activity by PI3K‐IN‐33 suppressed the inhibition of ferroptosis mediated by upregulation of PLK1 (Figure 6C). These results indicated that PLK1 reduced neuronal ferroptosis by activating the PI3K/AKT signalling pathway.

### 6.7 OTUD3 regulates PLK1 expression through deubiquitinating K48‐linked ubiquitination

**FIGURE 7:**
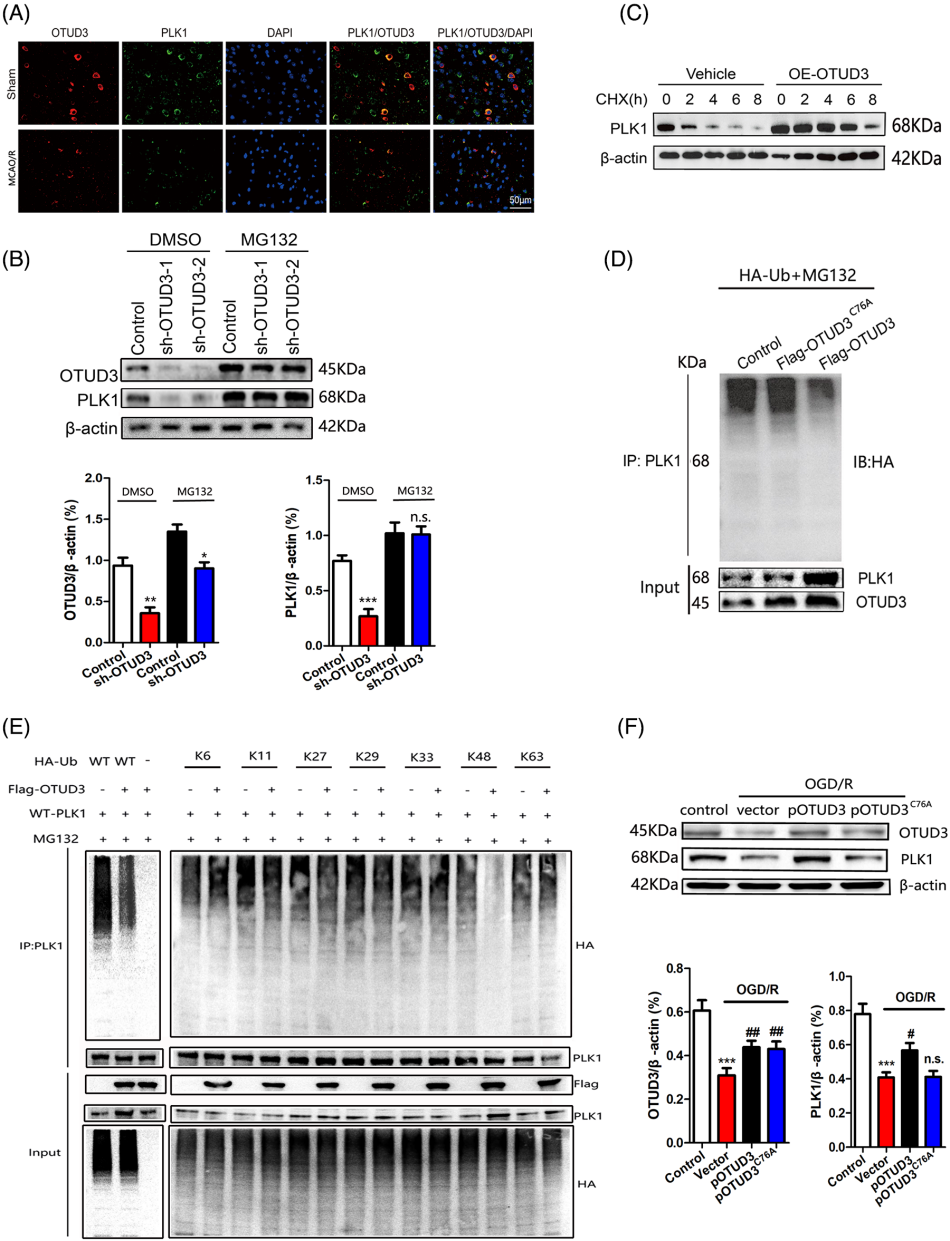
OTUD3 stabilizes PLK1 by deubiquitination. (A) Double immunofluorescence staining showed the localization of OTUD3 and PLK1 in mouse brain tissue sections. (B) Western blot was used to detect the protein expression of OTUD3 and PLK1 in different groups of 293T cells. Cells transfected with OTUD3 shRNA were treated with protease inhibitor MG132 (50 µM). (C) Western blot was used to analyse the expression of PLK1 protein in different groups of 293T cells. Cells transfected with OTUD3 overexpression plasmid were treated with CHX (10 µM) for 0, 2, 4, 6, and 8 h. (D) HA‐Ub plasmid was transfected into 293T cells, followed by Flag‐pCMV‐control, Flag‐pCMV‐OTUD3 or Flag‐pCMV‐OTUD3^C76A^ plasmids. Whole cell lysates were treated with MG132 for 8 h and collected for co‐immunoprecipitation with HA antibody. Western blot was used to detect the ubiquitination and protein expression of PLK1. (E) Effects of the indicated polyubiquitin on OTUD3‐mediated PLK1 ubiquitination. 293T cells were transfected with the indicated ubiquitin under MG132 treatment. IP analysis with anti‐HA antibody and immunoblotting with antibodies of anti‐Flag and anti‐HA. (F) The empty vector plasmid, pCMV‐OTUD3 or pCMV‐OTUD3^C76A^ plasmid were transfected into mouse cortical neurons and then OGD/R (90 min/24 h) was performed to establish the model. The protein expression levels of OTUD3 and PLK1 were detected by western blot (*n* = 6 in each group; ***p < .001 compared to Control; ^#^ *p* < .05 or ^##^ *p* < .01 compared to OGD/R + Vector; n.s., no significance compared to OGD/R + Vector; the two‐way ANOVA test, followed by the Bonferroni post hoc test). Control: normal 293T cell or normal mice cortical neurons. OGD/R+Vector: the neurons were transfected with empty vector plasmid and OGD/R model was established. OGD/R+pOTUD3: the OGD/R model was established after neurons were transfected with OTUD3 plasmid. OGD/R+pOTUD3^C76A^: the OGD/R model was established after neurons were transfected with OTUD3^C76A^ plasmid.

Using immunofluorescence colocalization, we found that OTUD3 and PLK1 could superpose with each other in the cytoplasm (Figure 7A), which provided spatial support for their interaction. To further explore whether OTUD3 affects the expression of PLK1 in a proteasome‐dependent manner, we treated 293T cells transfected with sh‐OTUD3 with the proteasome inhibitor MG132. In the DMSO group, compared to the Control group, the expression of PLK1 significantly decreased after the addition of OTUD3 shRNA. However, in the group treated with the proteasome inhibitor MG132, no significant reduction in PLK1 expression was observed after the addition of OTUD3 shRNA compared to the Control group. The results suggested that MG132 inhibited the downregulation of PLK1 mediated by sh‐OTUD3 (Figure 7B). When 293T cells were treated with the protein synthesis inhibitor cycloheximide CHX, overexpression of OTUD3 resulted in a significant increase in PLK1 protein stability (Figure 7C). We also transfected empty plasmid Flag‐pCMV‐control, OTUD3 full‐length plasmid Flag‐pCMV‐OTUD3 or OTUD3 deubiquitination enzyme site mutation plasmid Flag‐pCMV‐OTUD3^C76A^ plasmid into 293T cells, followed by treatment with a HA‐ubiquitin plasmid and MG132. Co‐IP experiments showed that compared with the MG132+Flag‐pCMV‐OTUD3^C76A^ group, the MG132+Flag‐pCMV‐OTUD3 group showed an increase in total PLK1 protein and a significant decrease in the ubiquitination level of PLK1 protein. Compared with those in the MG132+Flag‐pCMV‐control group, the ubiquitination levels of total PLK1 protein and PLK1 protein in the MG132+Flag‐pCMV‐OTUD3^C76A^ group did not change significantly (Figure 7D). These results indicated that OTUD3 upregulates PLK1 expression through deubiquitination modification. To determine the linkage type of the polyubiquitin chains that OTUD3 regulates PLK1, a series of Ub mutants were generated to preserve Lys on specific residues (Kx = K6, K11, K27, K29, K33, K48 and K63, with the other Lys residues mutated into Arginine, while wild type (WT) refers to the full‐length Ub plasmids without mutations). Overexpression of OTUD3 decreased K48‐linked polyubiquitination of PLK1, but not K6, K11, K27, K29, K33 and K63‐linked polyubiquitination (Figure 7E). We further confirmed that overexpression of OTUD3 reduced K48‐linked polyubiquitination of PLK1. Subsequently, we verified these results in OGD/R‐induced mouse cortical neurons, in which transfection with pCMV‐OTUD3 plasmid led to the upregulation of PLK1 protein expression, while transfection of pCMV‐OTUD3^C76A^ plasmid had no effect on PLK1 protein expression (Figure 7F). Taken together, these results indicate that OTUD3 stabilized PLK1 protein and promoted PLK1 expression through deubiquitination modification.

### 6.8 OTUD3 exerts neuroprotective effects in cerebral I/R by deubiquitination modification of PLK1

**FIGURE 8:**
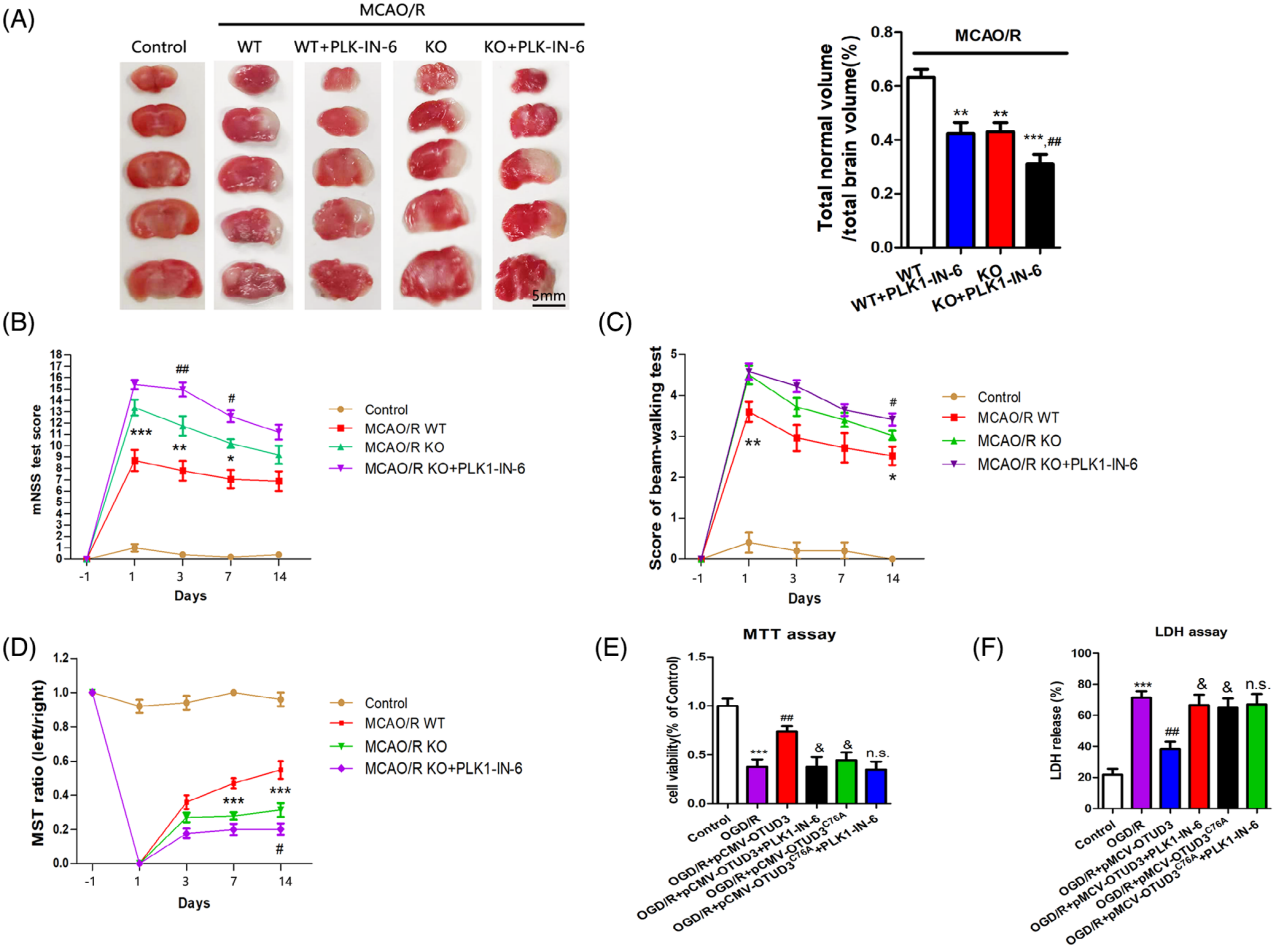
OTUD3 plays a neuroprotective role in cerebral ischaemia‐reperfusion (I/R) by modifying PLK1 by deubiquitination. (A) TTC staining results showed the cerebral infarct volume of each group. (B) The mNSS test shows the scores of each group at 1, 3, 7 and 14 days after MCAO/R injury. (C) Beam walking test scores for each group at 1, 3, 7 and 14 days after MCAO/R injury. (D) The MST test shows the scores of each group at 1, 3, 7 and 14 days after MCAO/R injury. (E) MTT assay showed the survival rate of neurons in each group. (F) LDH assay showed the release level of cytotoxic LDH in each group (*n* = 6 for each group; *p < .05, **p < .01 or ***p < .001 compared to Control or MCAO/R WT; ^#^ *p* < .05 or ^##^ *p* < .01 compared to MCAO/R KO or OGD/R; ^&^ *p* < .05 compared to OGD/R+pMCV‐OTUD3; n.s., no significance compared to OGD/R+pMCV‐OTUD3+PLK1‐IN‐6; the two‐way ANOVA test, followed by the Bonferroni post hoc test). Control: normal mice cortical neurons or OTUD3 wild‐type mice. MCAO/R+WT: OTUD3 wild‐type mice middle cerebral artery occlusion 90 min/reperfusion 24 h. MCAO/R+WT+PLK1‐IN‐6: The MCAO/R model was established in OTUD3 wild‐type mice by intraventricular injection of PLK1‐IN‐6. MCAO/R+KO: OTUD3 knockout mice middle cerebral artery occlusion 90 min/reperfusion 24 h. MCAO/R+KO+PLK1‐IN‐6: The MCAO/R model was established in OTUD3 knockout mice by intraventricular injection of PLK1‐IN‐6. OGD/R: neurons transferred to a deoxygenated, glucose‐free extracellular solution for 90 min/restore oxygen and glucose. OGD/R+pCMV‐OTUD3: The OGD/R model was established after neurons were transfected with OTUD3 plasmid. OGD/R+ pCMV‐OTUD3^C76A^: The OGD/R model was established after neurons were transfected with OTUD3^C76A^ plasmid. OGD/R+pCMV‐OTUD3+PLK1‐IN‐6: The OGD/R model was established after neurons were transfected with OTUD3 plasmid and PLK1‐IN‐6. OGD/R+ pCMV‐OTUD3^C76A^+ PLK1‐IN‐6: The OGD/R model was established after neurons were transfected with OTUD3^C76A^ plasmid and PLK1‐IN‐6.

We investigated whether OTUD3 could exert neuroprotective effects in cerebral I/R by deubiquitination modification of PLK1. We used OTUD3 knockout mice to construct an MCAO/R model and the PLK1‐specific inhibitor PLK1‐IN‐6 was injected intracerebroventricularly. The results of TTC staining experiments showed that OTUD3 knockout aggravated cerebral infarction in MCAO/R mice, and treatment with PLK1‐IN‐6 resulted in a further increase in cerebral infarction volume in mice mediated by OTUD3 deletion (Figure 8A). Subsequently, we performed a series of animal behavioural tests and found that compared with the wild‐type mouse group, OTUD3 knockout mice scored higher in the mNSS test and Beamwalking test on days 1, 3, 7 and 14, and lower in the MST test on days 3, 7 and 14. OTUD3 knockout mice that were injected with PLK1‐IN‐6 scored higher in the mNSS test and Beamwalking test on days 3, 7 and 14, and lower in the MST test, compared with OTUD3 knockout mice that were not injected with PLK1‐IN‐6 (Figure 8B–D). These results indicated that OTUD3 knockout aggravated cerebral I/R injury, and inhibition of PLK1 further exacerbated brain injury, which suggests that PLK1 is involved in regulating the neuroprotective effects mediated by OTUD3 in cerebral I/R.

**FIGURE 9:**
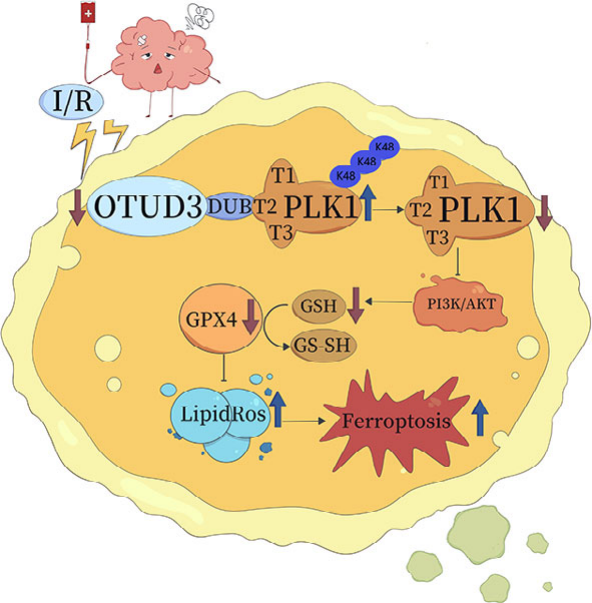
Figure of the mechanism by which OTUD3 upregulates PLK1 by deubiquitination to reduce ferroptosis after cerebral ischaemia‐reperfusion injury.

Next, we transfected either OTUD3 full‐length plasmid Flag‐pCMV‐OTUD3 or OTUD3 deubiquitination enzyme site mutation plasmid Flag‐pCMV‐OTUD3^C76A^ into primary cortical neurons from mice and treated them with PLK1‐IN‐6, a specific inhibitor of PLK1, to construct an OGD/R injury model. MTT and LDH experiments showed that overexpression of OTUD3 increased neuronal survival and reduced the release of cytotoxic LDH after I/R. Following overexpression of OTUD3^C76A^, there was no significant change in I/R‐induced neuronal survival and LDH release. Moreover, inhibition of PLK1 activity suppressed the effect of OTUD3 overexpression on neuronal survival and reducing the release of LDH. Compared with the OGD/R+pCMV‐OTUD3+PLK1‐IN‐6 group, the OGD/R+pCMV‐OTUD3^C76A^+PLK1‐IN‐6 group had no significant changes in the neuronal survival and LDH release (Figure 8E,F). The results showed that inhibition of PLK1 activity inhibited the neuroprotective effect of OTUD3 through deubiquitination of PLK1. These results suggest that OTUD3 exerts neuronal protective effects by deubiquitination of PLK1. Decreased expression of OTUD3 after cerebral I/R resulted in increased PLK1 ubiquitination, degradation of PLK1 and decreased PLK1 protein expression. In turn, this inhibited the activation of the PI3K/AKT signalling pathway, reduced GSH content, inhibited the activity of GPX4 and led to an increase in the generation of ROS, which ultimately contributed to the occurrence of ferroptosis in neuronal cells (Figure 9).

## 7 DISCUSSION

There are approximately more than 100 DUBs in the human proteome, grouped into six families by protein structure and molecular features: ubiquitin‐specific proteases, ovarian tumour proteases, ubiquitin c‐terminal hydrolase (UCHs), Jab1/MPN domain associated metalloisopeptidase, Machado‐josephin domain protease and Motif‐interacting with ubiquitin‐containing novel DUB family. Liu H et al. reported that UCHL1, a deubiquitinating enzyme, reduced neuronal damage in mice with ischaemic stroke.^11^ Kahles et al. found that deubiquitinating enzyme could protect proteins from oxidative stress damage and reduce brain damage caused by ischaemic stroke by maintaining protein integrity by regulating the activity of deubiquitinating enzyme.^12^ Chen et al. recently found that the deubiquitinating enzyme USP30 reduces neuronal damage induced by cerebral ischaemia by maintaining mitochondrial morphology through deubiquitinating modification.^32^ Ganjam et al. found that downregulation of the deubiquitinating enzyme CYLD can increase RIP1 ubiquitination. Preventing the formation of RIP1/RIP3 complex reduces neuron death.^33^ These studies suggest that deubiquitination modification affects disease prognosis through key factors involved in the regulation of the pathological development process of cerebral I/R injury. Our team found that the deubiquitination enzyme OTUD3 plays an important role in cerebral I/R injury, and the decreased expression of OTUD3 protein after cerebral I/R injury leads to the reduction of PLK1 protein level, which inhibits the activation of PI3K/AKT signalling pathway and ultimately promotes neuron ferroptosis. Hou et al. discovered that OTUD3 reduces ferroptosis in hippocampal neurons by stabilizing IRP2.^17^ This finding is consistent with our research results. The inspiration for this article comes from previous studies that demonstrated OTUD3 stabilizes IRP2 to prevent Parkinson's disease.^34^ By constructing an I/R model in hippocampal neurons and overexpressing OTUD3 or IRP2 via lentiviral transduction, the effects on hippocampal neurons were investigated. However, the experimental methods used by Hou et al. are relatively limited. Moreover, this approach has several potential confounding factors, including transduction efficiency, success rate of model establishment, variations in experimental conditions and cell viability. These factors can introduce significant variability, limiting the reliability of the conclusions. Therefore, building on their previous research, our team adopted a combination of techniques, including transgenic knockout, lentiviral transduction, plasmid transfection and the use of inhibitors, to validate our findings in both in vivo and in vitro mouse cortical neurons. This multi‐layered approach minimized experimental errors and enhanced the reliability of our conclusions. Mechanistically, we conducted further research. Through co‐immunoprecipitation‐mass spectrometry experiments, we discovered that OTUD3 can specifically interact with PLK1.

Polo‐like kinases are a conserved class of serine/threonine protein kinases. It has been found that there are five subtypes of PLK1, PLK2 (SNK), PLK3 (FNK or PRK), PLK4 (SAK) and PLK5 in mammals, which play an important role in the regulation of cell mitotic cycle and growth.^35^ PLK1 has been associated with the occurrence and growth of malignant tumours in many studies, and PLK1 can promote tumour cell growth.^36^, ^37^ In the regulation of the cell cycle, PLK1 kinase activity plays a central role by phosphorylating downstream target proteins to regulate the G2/M checkpoint transition and mitotic progression. The significance of PLK1 kinase activity not only lies in its precise control of cell division but also in its dual role in maintaining genomic integrity.^35^ As a key driver of mitosis, PLK1 dynamically regulates core processes such as spindle assembly, sister chromatid separation and cytokinesis through phosphorylation. Dysregulation of its activity not only directly leads to chromosomal segregation errors and micronucleus formation but may also exacerbate oncogenic risk by interfering with DNA damage repair pathways, making it a highly promising molecular target in cancer therapy.^35^, ^36^, ^37^ Co‐immunoprecipitation and mass spectrometry showed that OTUD3 interacted with PLK1 and then truncated PLK1 into three parts. Through co‐immunoprecipitation and ubiquitination experiments, it was found that OTUD3 deubiquitinated PLK1‐T2 to reduce the ubiquitination and degradation of PLK1 and increase the accumulation of PLK1 protein in cells. The results suggest that decreased OTUD3 after I/R injury indirectly leads to decreased PLK1 levels. Similar results were obtained by single‐cell sequencing, which showed that PLK1 level was significantly reduced in mouse neurons after cerebral ischaemia, which was consistent with the previous findings. Subsequently, we verified this in mouse cortical neurons. After transfection of full‐length OTUD3 plasmid into cultured neurons in vitro, the expression of PLK1 protein was significantly upregulated and the ubiquitination level was decreased. There was no significant change in PLK1 protein and ubiquitination level after transfection with OTUD3 deubiquitination site mutant plasmid. These results suggest that OTUD3 increases PLK1 stability and upregulates PLK1 protein expression through the action of deubiquitinating enzyme. We further found that OTUD3 modified PLK1 through deubiquitinating K48‐linked ubiquitination. This result further confirmed that overexpression of OTUD3 reduced K48‐linked polyubiquitination of PLK1.

Previous studies have shown that PLK1 is closely related to cell ferroptosis, and PLK1 knockout promotes ferroptosis by inhibiting the pentose phosphate pathway in ESCC.^20^ The same results were obtained in the present study. In OGD/R‐injured mouse cortical neurons, the addition of PLK1 inhibitor increased the ferroptosis product and the level of ferroptosis. Therefore, we further investigated the effect of OTUD3 knockout/upregulation on ferroptosis after I/R injury. The results showed that knockout OTUD3 aggravated I/R‐induced ferroptosis, and upregulation of OTUD3 significantly reduced I/R‐induced ferroptosis. These results suggest that OTUD3 plays a neuroprotective role in cerebral I/R injury by inhibiting neuron ferroptosis.

We subsequently added PLK1 inhibitor to OTUD3 knockout mice and found that the level of ferroptosis was further increased. Our previous study showed that OTUD3 overexpression could protect neurons from I/R‐induced ferroptosis. However, inhibition of PLK1 attenuated this neuroprotective effect. Interestingly, we found that overexpression of PLK1 reduced ferroptosis by activating PI3K/AKT signalling pathway in mouse cortical neurons injured by OGD/R, and the ability of PLK1 to reduce ferroptosis was significantly impaired by inhibition of PI3K activity. Finally, a series of animal behavioural experiments and cell survival experiments were used to verify the same experimental results. The results showed that OTUD3 knockout aggravated I/ R‐induced brain damage and neurons death, and inhibition of PLK1 further aggravated the damage. After inhibiting the activity of OTUD3 deubiquitinating enzyme, the neuroprotective effect of upregulated OTUD3 was significantly weakened. Compared with the OGD/R+pCMV‐OTUD3+PLK1‐IN‐6 group, the OGD/R+pCMV‐OTUD3^C76A^+PLK1‐IN‐6 group had no significant changes in the survival rate and LDH release. The results showed that inhibition of PLK1 activity inhibited the neuroprotective effect of OTUD3 through deubiquitination of PLK1. Compared with the OGD/R+pCMV‐OTUD3^C76A^ group, the survival rate of neurons in the OGD/R+pCMV‐OTUD3^C76A^+PLK1‐IN‐6 group was slightly decreased, and LDH release was relatively increased, but there was no statistical significance. This is a provocative result, indicating that deubiquitination of PLK1 by OTUD3 is one of the major ways of PLK1 degradation during OGD/R injury, but there are other factors affecting the activity and function of PLK1 in the body or OTUD3 can act through other pathways. OTUD3 has been shown to reduce ferroptosis by stabilizing IRP2.^17^ PLK1 may influence ferroptosis through its effects on IRP2. This is because PLK1 is involved in cellular stress responses and can regulate the intracellular redox state by modulating the antioxidant system.^38^ Oxidative stress can affect the stability of the oxidation‐sensitive protein IRP2, with high levels of oxidative stress leading to its degradation.^39^ Therefore, PLK1 may indirectly influence the stability or activity of IRP2 by regulating the levels of oxidative stress. In subsequent studies, the use of primary neurons from PLK1 knockout mice to validate the above experiments may obtain more definitive research results.

Taken together, our findings provide evidence that OTUD3 plays a neuroprotective role in cerebral I/R injury by upregulating PLK1 through deubiquitination and reducing ferroptosis. These findings provide key experimental and theoretical evidence for the identification of novel therapeutic targets for ischaemic stroke.

## AUTHOR CONTRIBUTIONS

JC and XHQ partook in thecell experimentation andcell culture. YQF and JC were primarily responsible for the design and writing of the manuscript. QT contributed to ubiquitination experiments and fluorescence staining experiments. HRL and LQW were involved in the in vivo experimentation and in feeding the knockout mice. HXJ contributed to animal behavioral experiments and cell phenotype experiments. LQW and ZBC participated in laying out the experimental method guidance and the design of the manuscript. ZBC and YQF revised the manuscript. All the authors read, revised, and approved the submitted version of the manuscript.

## CONFLICT OF INTEREST STATEMENT

The authors declare no conflicts of interest.

## ETHICS STATEMENT

All animal use and testing protocols were approved and implemented in accordance with the guidelines of the Institutional Animal Protection and Use Committee and the Animal Protection and Ethics Committee of Wuhan University School of Medicine (approval no. WDRM‐20200301C). The samples were collected and processed by random sampling method. All animal‐related experiments were conducted in accordance with the Animal Research: In Vivo Laboratory Report guidelines.

## Supporting information



Supporting Information

Supporting Information

Supporting Information

Supporting Information

Supporting Information

Supporting Information
