# Supplementary figures and images for: The deubiquitinase OTUD3 plays a neuroprotective role by reducing ferroptosis induced by cerebral ischaemia reperfusion via stabilizing PLK1 via deubiquitination
Source: Clin Transl Med. 2025 Jun 3;15(5):e70347. doi: 10.1002/ctm2.70347 (PMC12134399; doi:10.1002/ctm2.70347)

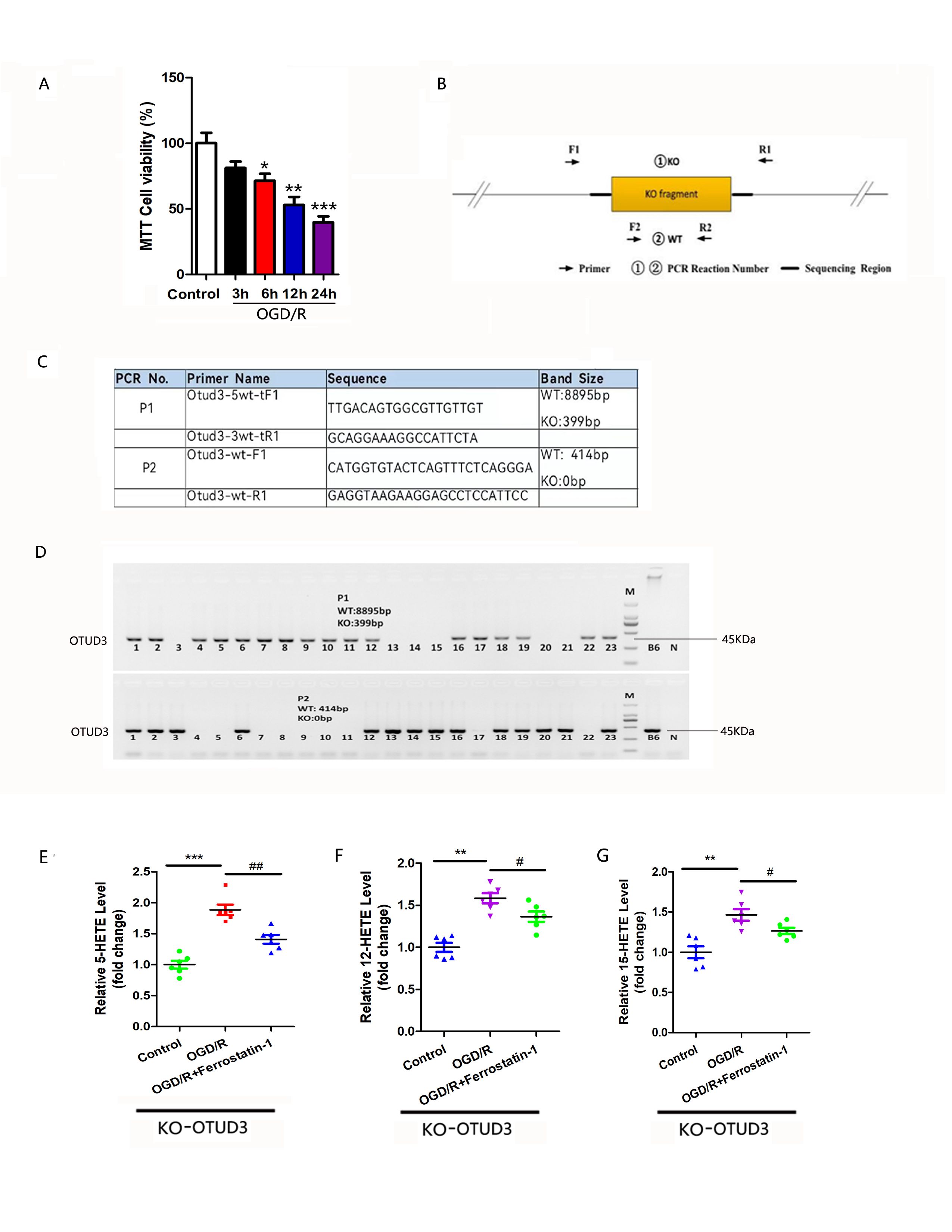

Supplement: Supplementary file 1 — Supporting Information [file CTM2-15-e70347-s003.tif]

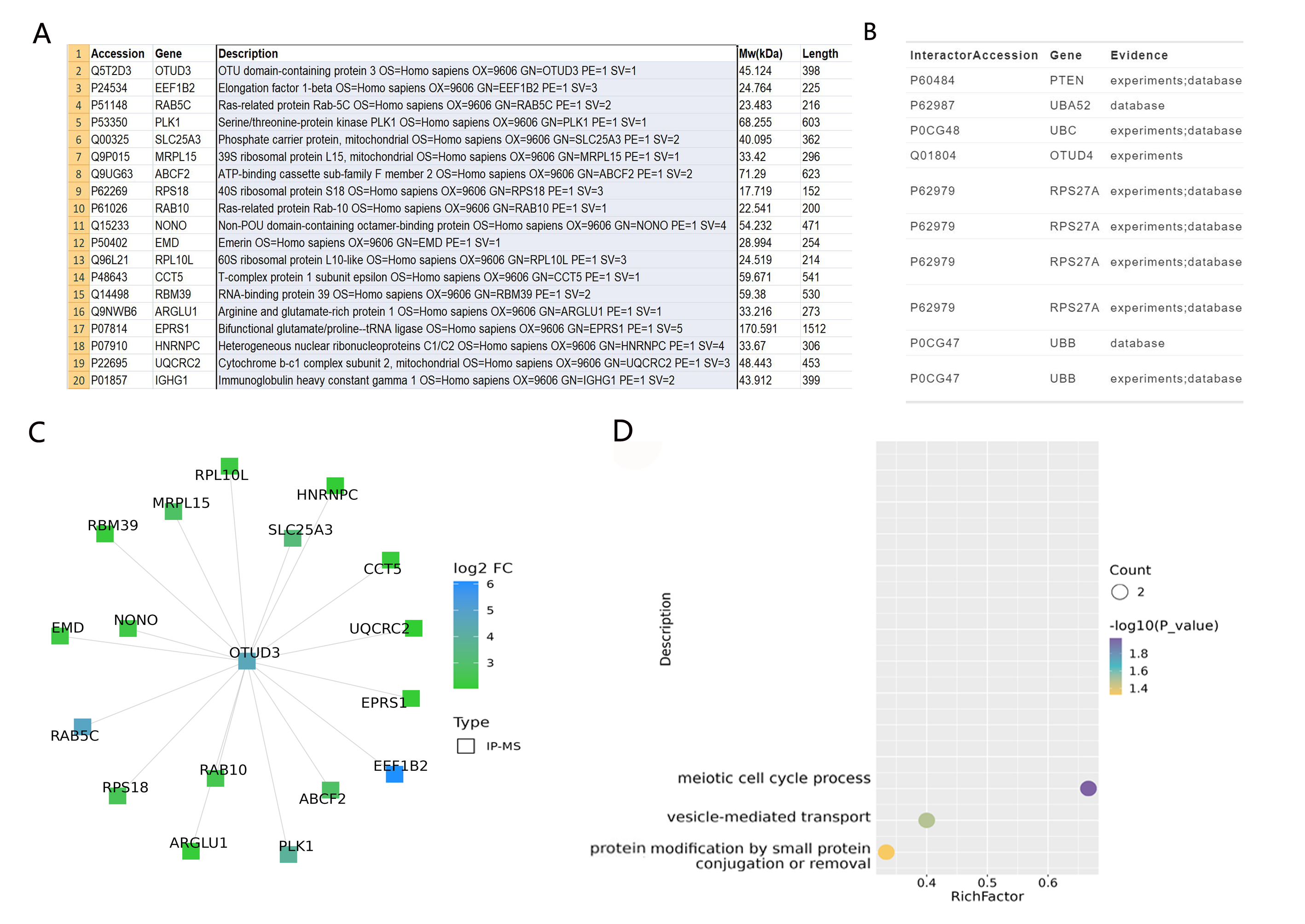

Supplement: Supplementary file 2 — Supporting Information [file CTM2-15-e70347-s002.tif]

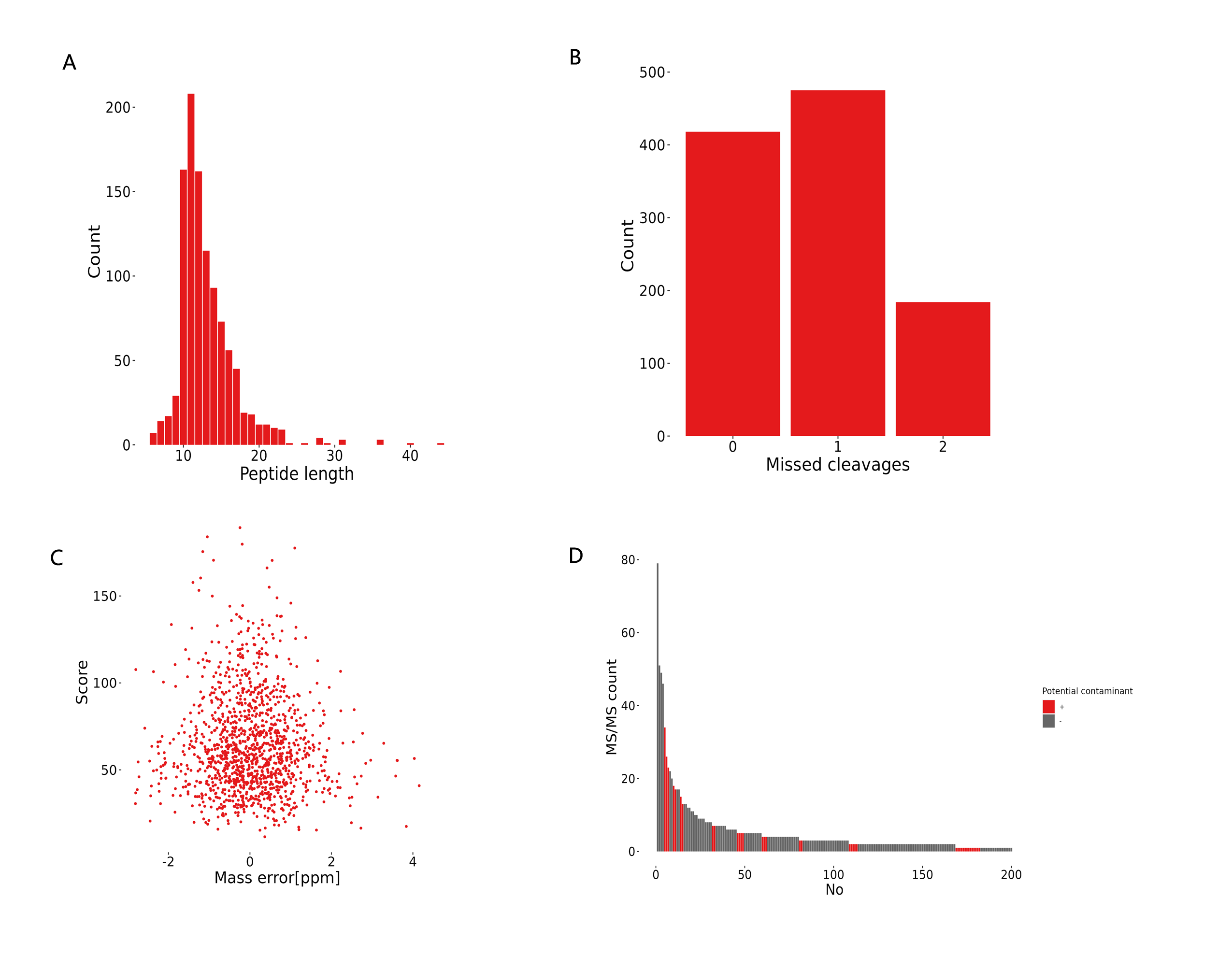

Supplement: Supplementary file 3 — Supporting Information [file CTM2-15-e70347-s005.tif]

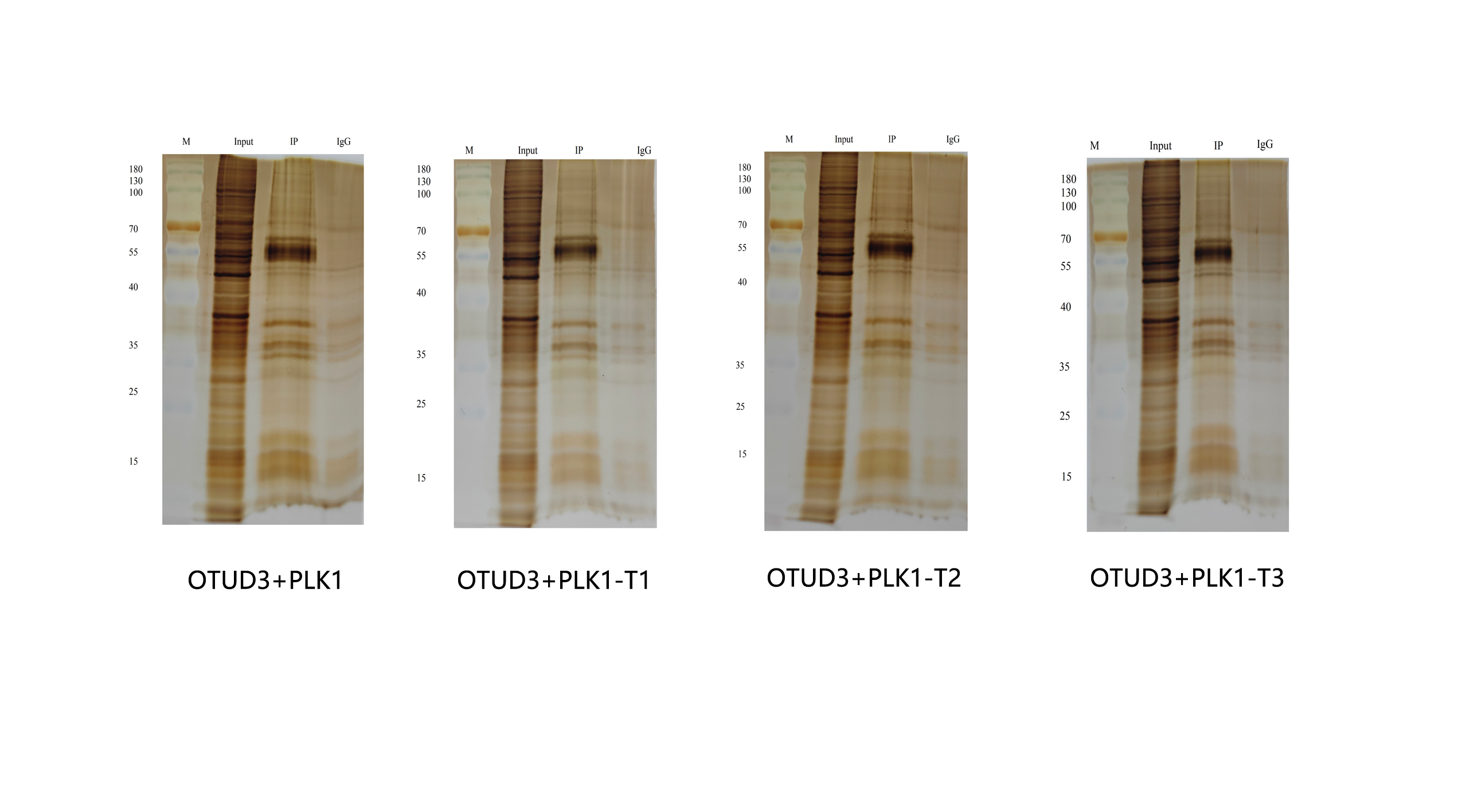

Supplement: Supplementary file 4 — Supporting Information [file CTM2-15-e70347-s006.tif]

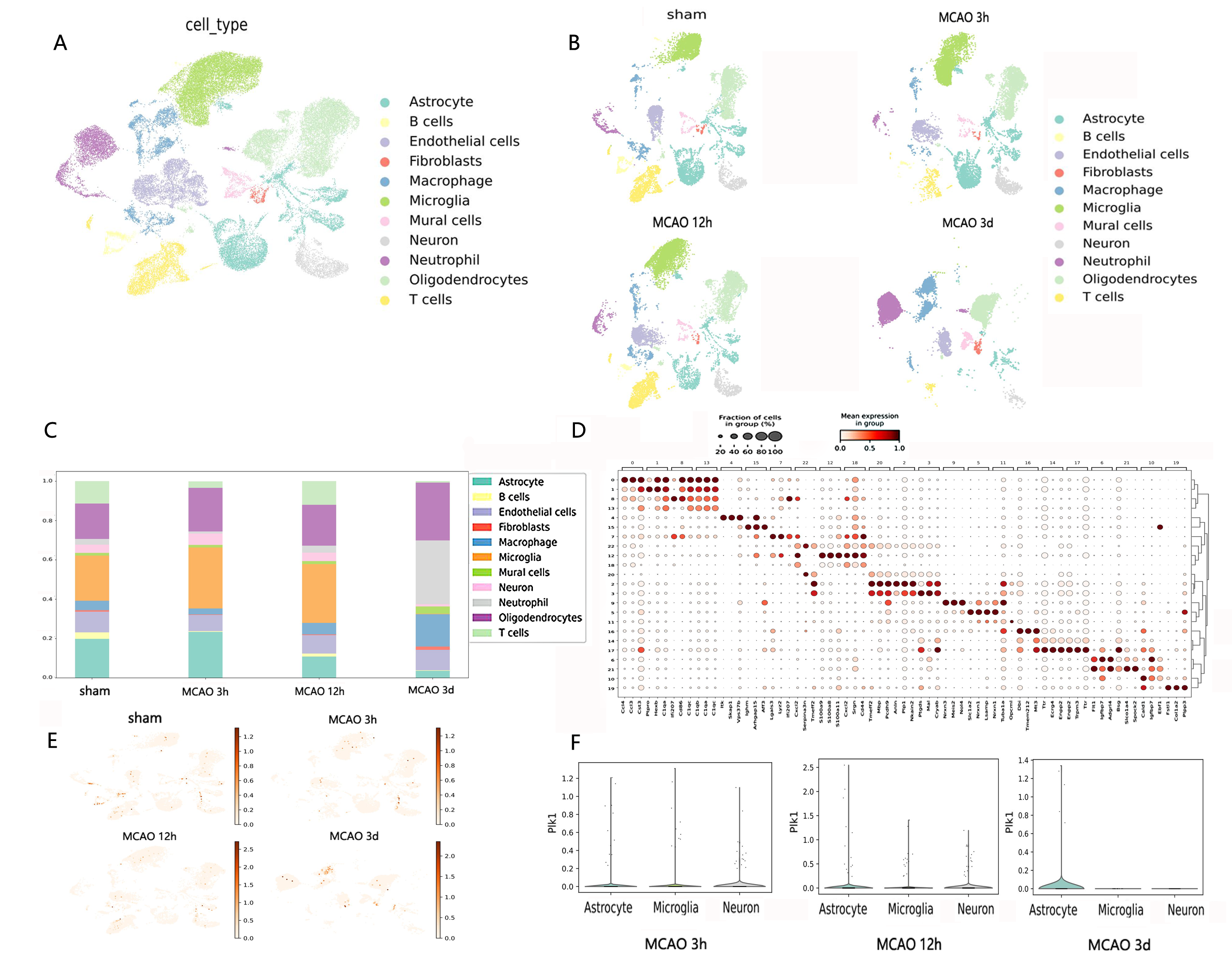

Supplement: Supplementary file 5 — Supporting Information [file CTM2-15-e70347-s001.tif]

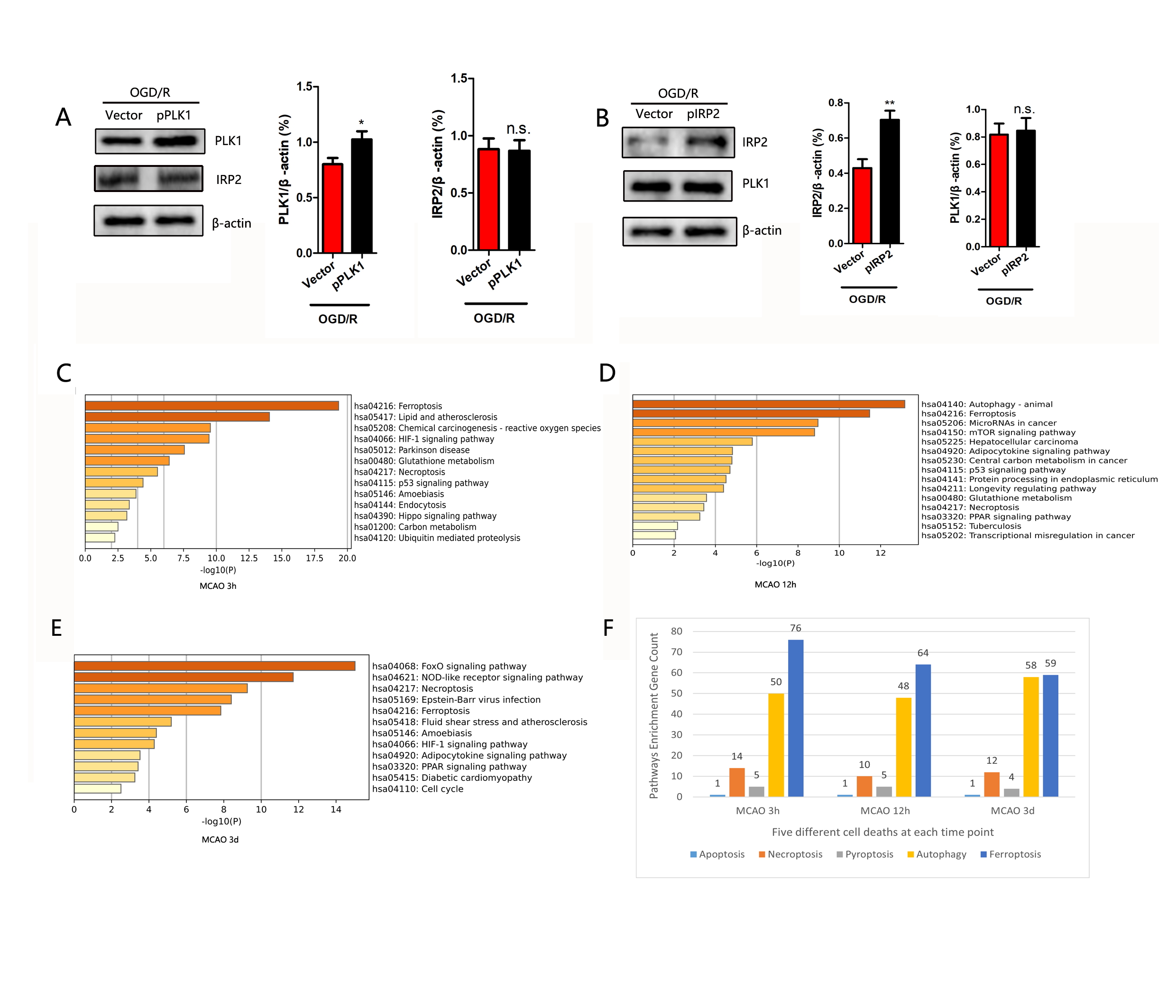

Supplement: Supplementary file 6 — Supporting Information [file CTM2-15-e70347-s004.tif]
